# Perfluorocycloparaphenylenes

**DOI:** 10.1038/s41467-022-31530-x

**Published:** 2022-06-28

**Authors:** Hiroki Shudo, Motonobu Kuwayama, Masafumi Shimasaki, Taishi Nishihara, Youhei Takeda, Nobuhiko Mitoma, Takuya Kuwabara, Akiko Yagi, Yasutomo Segawa, Kenichiro Itami

**Affiliations:** 1 Graduate School of Science, Nagoya University, Nagoya, 464-8602 Japan; 2 JST, ERATO, Itami Molecular Nanocarbon Project, Nagoya University, Nagoya, 464-8602 Japan; 3 Institute of Transformative Bio-Molecules (WPI-ITbM) Nagoya University, Nagoya, 464-8602 Japan; 4 Institute of Advanced Energy, Kyoto University, Kyoto, 611-0011 Japan; 5 Department of Applied Chemistry, Graduate School of Engineering, Osaka University, Yamadaoka 2-1, Suita, Osaka, 565-0871 Japan; 6 RIKEN Center for Emergent Matter Science, Wako, 351-0198 Japan; 7 Institute for Molecular Science, Myodaiji, Okazaki, 444-8787 Japan; 8 Department of Structural Molecular Science, SOKENDAI (The Graduate University for Advanced Studies), Myodaiji, Okazaki, 444-8787 Japan

**Keywords:** Organic chemistry, Chemical synthesis, Supramolecular chemistry

## Abstract

Perfluorinated aromatic compounds, the so-called perfluoroarenes, are widely used in materials science owing to their high electron affinity and characteristic intermolecular interactions. However, methods to synthesize highly strained perfluoroarenes are limited, which greatly limits their structural diversity. Herein, we report the synthesis and isolation of perfluorocycloparaphenylenes (PFCPPs) as a class of ring-shaped perfluoroarenes. Using macrocyclic nickel complexes, we succeeded in synthesizing PF[*n*]CPPs (*n* = 10, 12, 14, 16) in one-pot without noble metals. The molecular structures of PF[*n*]CPPs (*n* = 10, 12, 14) were determined by X-ray crystallography to confirm their tubular alignment. Photophysical and electrochemical measurements revealed that PF[*n*]CPPs (*n* = 10, 12, 14) exhibited wide HOMO–LUMO gaps, high reduction potentials, and strong phosphorescence at low temperature. PFCPPs are not only useful as electron-accepting organic materials but can also be used for accelerating the creation of topologically unique molecular nanocarbon materials.

## Abstract (web summary)

Synthetic methods for the preparation of perfluorinated aromatic compounds are desirable in materials science. Here, the authors synthesize perfluorocycloparaphenylenes, fully fluorinated carbon nanorings, through a nickel-mediated one-pot method.

## Introduction

**Fig. 1:**
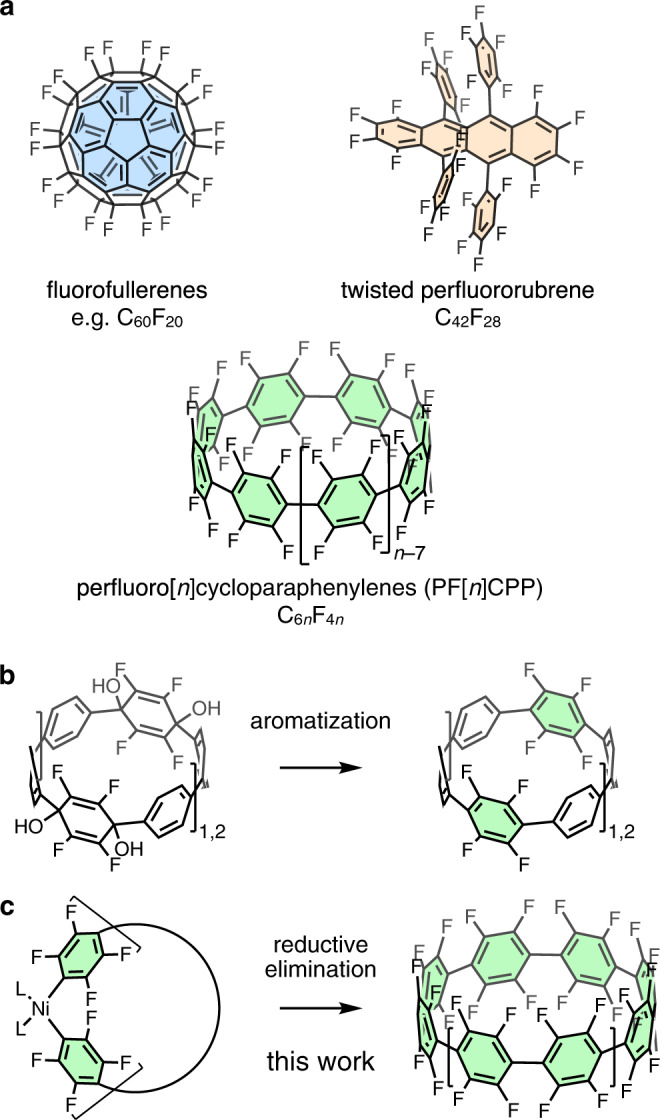
Fluorocarbon molecules. **a** Structures of fluorofullerenes, perfluororubrene, perfluorocoranullene (top), and perfluorocycloparaphenylenes (PFCPPs) (bottom). **b** A synthetic scheme of previously reported partially fluorinated CPPs. **c** The synthetic strategy for PFCPPs (this work).

Organic fluorine compounds have found widespread applications in pharmaceutical, agricultural, and materials science^1–5^. The introduction of fluorine into organic molecules often strongly affects their properties, including their polarity, solubility, and lipophilicity. Among many organic fluorine-containing compounds, fluorinated arenes are used as semiconductors, light-emitting materials, and liquid crystals^6^. Owing to its negative inductive effect, the incorporation of fluorine into materials leads to a decrease in orbital energy. As such, it is important to develop synthetic methods that provide access to aromatic molecules containing many C–F bonds^7–9^, and the extreme targets of the research field are aromatic molecules wherein all hydrogen atoms are replaced with fluorine atoms, i.e., perfluoroarenes^10–14^. However, methods to synthesize strained perfluoroarenes remain very limited. It is known that various fluorinated fullerenes (Fig. 1a, top left) can be obtained from the addition of fluorine to the unsaturated bonds of fullerenes, but these are virtually the only examples of highly strained perfluoroarenes^15^. Although Suzuki and co-workers have shown that perfluororubrene (Fig. 1a, top right) possesses a twisted structure with a slight strain^16^, a method to apply more strain to perfluoroarenes has not yet been reported.

Perfluorocycloparaphenylenes (PFCPPs) are a class of highly strained ring-shaped perfluoroarenes (Fig. 1a, bottom) in which all hydrogen atoms of the corresponding cycloparaphenylenes (CPPs)^17–19^ are replaced with fluorine atoms. This replacement of hydrogen with fluorine can be expected to result in a significant change of the structural and electronic properties of the PFCPPs^20^. Two major methods for the synthesis of CPPs have been reported: (i) converting C6 units, such as cyclohexane and cyclohexadiene, of macrocyclic precursors into benzene rings^21,22^, and (ii) reductive elimination from macrocyclic metal–arene complexes (metal = Pt, Ni, Au)^23–25^. However, even partially fluorinated CPPs (F_8_[6]CPP, F_12_[9]CPP, F_8_[12]CPP, F_8_[10]CPP, F_8_[12]CPP) synthesized by the groups of Yamago and Jasti^26–28^ require multiple steps (method (i), Fig. 1b), and there has no successful synthesis of CPP derivatives from *ortho*-functionalized aryl groups by the Pt method^17–19^. We hypothesized that PFCPPs can be obtained in one-pot based on method (ii). Considering that the reductive elimination of perfluorobiphenyl occurs from the stable (2,2′-bipyridyl)Ni(C_6_F_5_)_2_ complex promoted by acids or oxidants^29,30^, Ni might be a suitable metal for the construction of macrocyclic precursors for PFCPPs (Fig. 1c).

Herein, we report the synthesis and isolation of PFCPPs. Using macrocyclic nickel complexes, PF[*n*]CPPs (*n* = 10, 12, 14, 16) were obtained in one-pot without using noble metals. The molecular structures of PF[*n*]CPPs (*n* = 10, 12, 14) were determined by X-ray crystallography to confirm their structures and tubular alignment. PF[*n*]CPPs (*n* = 10, 12, 14) exhibited wide HOMO–LUMO gaps, high reduction potentials, and strong phosphorescence at low temperature.

## Results and discussion

### Synthesis of PFCPPs

**Fig. 2:**
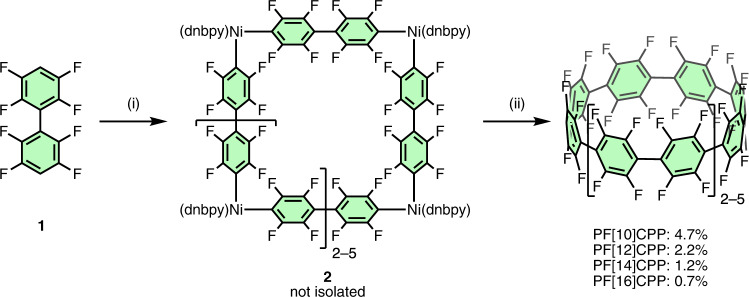
Synthesis of PFCPPs. Reaction conditions: (i) LDA, Ni(dnbpy)Br_2_, THF, −78 °C, 30 min; (ii) DDQ, *m*-xylene, 130 °C, 5 h. LDA = lithium diisopropylamide; dnbpy = 4,4′-di-*n*-nonyl-2,2′-bipyridyl; DDQ = 2,3-dichloro-5,6-dicyano-*p*-benzoquinone.

Our synthetic route to PFCPPs is outlined in Fig. 2. Starting from 2,3,5,6,2′,3′,5′,6′-octafluorobiphenyl (**1**), deprotonation by lithium diisopropylamide (LDA) and subsequent transmetallation to Ni(dnbpy)Br_2_ (dnbpy = 4,4′-di-*n*-nonyl-2,2′-bipyridyl) produced a mixture of macrocyclic complex **2**. After evaporation of the solvent and replacing it with *m*-xylene, 2,3-dichloro-5,6-dicyano-*p*-benzoquinone (DDQ) was added and the resulting mixture was stirred at 130 °C for 5 h to promote the reductive elimination of aryl–aryl bonds from Ni. By the purification with silica gel chromatography and preparative recycle GPC, PF[*n*]CPPs (*n* = 10, 12, 14, 16) were obtained in 4.7%, 2.2%, 1.2%, and 0.7% yield, respectively. These PF[*n*]CPPs are highly strained perfluoroarenes, as evident from their high strain energies of 60.2 (*n* = 10), 49.9 (*n* = 12), 42.6 (*n* = 14), and 37.2 kcal·mol^–1^ (*n* = 16) estimated by density-functional theory (DFT) calculations (for details, see Supplementary Fig. 10 in Supplementary Information (SI)). Considering that PF[*n*]CPPs were not obtained when 2,2′-bipyridyl or 4,4′-di-*t*-butyl-2,2′-bipyridyl was used, the *n*-nonyl groups of the dnbpy ligand should be crucial for preventing the precipitation of intermediates. For each PF[*n*]CPP (*n* = 10, 12, 14, 16), one singlet signal was observed in the ^19^F NMR spectra at −138.25 (*n* = 10), −138.50 (*n* = 12), −138.64 (*n* = 14), and −138.84 ppm (*n* = 16), where the trend to shift resonances to lower magnetic field with increasing ring size is similar to the case of ^1^H NMR chemical shifts of [*n*]CPPs^17^. Two singlet signals observed in the ^13^C{^19^F} NMR spectra also agreed with the high-symmetric structures of PFCPPs. The corresponding high-resolution mass spectra were recorded using the negative mode of the LDI-TOF MS (laser desorption/ionization time-of-flight mass spectrometry) technique. IR and Raman spectra of PFCPPs are in good agreement with the calculated spectra by B3LYP/6-31G(d) level of theory (see Supplementary Fig. 7). Thus, these compounds were identified based on spectral measurements.

### Structures of PFCPPs

**Fig. 3:**
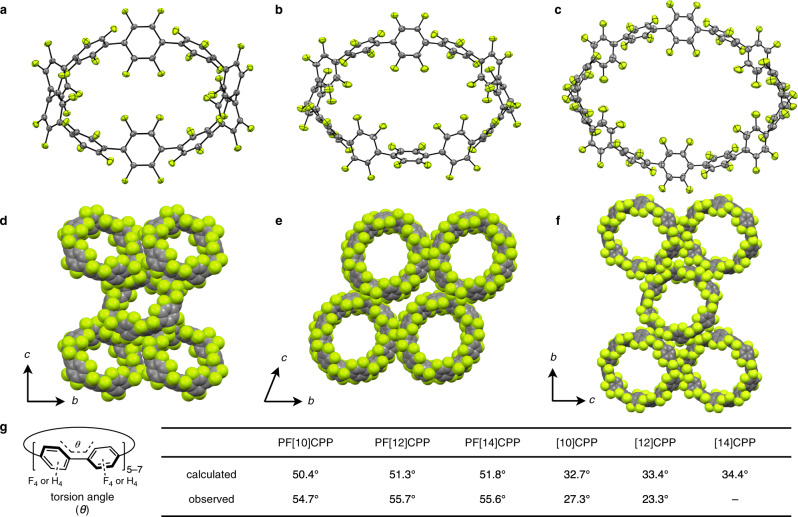
X-ray crystal structures of PF[n]CPPs (*n* = 10, 12, 14). **a**, **b**, **c** Structures of PF[10]CPP (**a**), PF[12]CPP (**b**), and PF[14]CPP (**c**) with thermal ellipsoids at 50% probability. Solvent molecules are omitted for clarity. **d**, **e**, **f** Packing structures of PF[10]CPP (**d**), PF[12]CPP (**e**) and PF[14]CPP (**f**); gray: carbon; green: fluorine. Solvent molecules are omitted for clarity. **g** The dihedral angles (*θ*) around the C–C single bonds of PFCPPs and corresponding CPPs^31, 32^. Calculations were performed at the B3LYP/6-31G(d) level of theory. Dihedral angles obtained from X-ray crystallography are averaged.

The structures of PF[*n*]CPPs (*n* = 10, 12, 14) were successfully determined by X-ray crystallography. Single crystals of PF[10]CPP, PF[12]CPP, and PF[14]CPP were obtained from THF, hexafluorobenzene/*n*-hexane, and chloroform/*n*-pentane solutions, respectively. As shown in Fig. 3a–c, perfluoroarene structures with CPP skeletons (F_40_[10]CPP, F_48_[12]CPP, F_56_[14]CPP) were unambiguously confirmed. For PF[10]CPP, THF used for recrystallization is contained within the ring, despite the fact that these molecules are strongly disordered, whereas PF[12]CPP contains four molecules of hexafluorobenzene inside and outside the rings. The crystal packing of these PFCPPs is shown in Fig. 3d–f. In PFCPPs, the ring cavities are aligned along the *a*-axis. This result stands in contrast to the behavior of [*n*]CPPs of the same size (*n* = 10, 12), which show herringbone-like packing^31,32^, indicating that the influence of fluorine atoms on the molecular alignment in crystal state is significant. Similar tubular packing was also found for partially fluorinated CPPs^26–28^. The torsion angles between pairs of benzene rings are summarized in Fig. 3g. The averaged dihedral angles observed in X-ray crystallography (PF[10]CPP: 54.7°; PF[12]CPP: 55.7°; PF[14]CPP: 55.6°) and those obtained from DFT optimizations at the B3LYP/6-31 G(d) level of theory (PF[10]CPP: 50.4°; PF[12]CPP: 51.3°; PF[14]CPP: 51.8°) are higher than those of [10]- and [12]CPP (calculated: ~33°; observed: 23–27°)^31–33^, which is most likely caused by the steric repulsion between fluorine atoms. The interaction between PF[10]CPP and fullerene C_60_ in deuterated chloroform solution was observed (Supplementary Fig. 14)^34–36^, while the stoichiometry of supramolecular complexes could not be determined by titration experiment because of low solubility of [10]PFCPP and its C_60_ complex.

### Electronic properties of PFCPPs

**Fig. 4:**
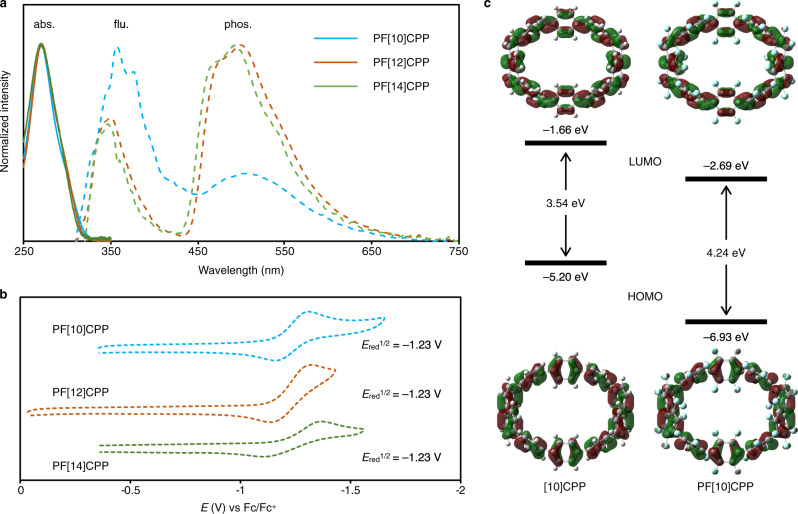
Photophysical properties of PF[n]CPPs (*n* = 10, 12, 14). **a** UV–Vis absorption spectra of dichloromethane solutions of PFCPPs at room temperature (solid lines), and photoluminescence spectra of PFCPPs in ethanol grass at 77 K upon excitation at 270 nm (dashed lines); blue: PF[10]CPP; red: PF[12]CPP; green: PF[14]CPP. **b** Cyclic voltammograms of PFCPPs in acetonitrile (supporting electrolyte: [*n*-Bu_4_N][PF_6_]; scan rate: 0.1 V s^−1^). **c** Frontier molecular orbitals (isovalue: 0.02) and their energies (eV) of [10]CPP and PF[10]CPP calculated by B3LYP/6-31G(d) level of theory. Fc ferrocene.

In order to investigate the effect of C–F bonds on the π-electrons in PFCPPs, optical and electrochemical measurements as well as DFT calculations were carried out. The PFCPPs showed absorption in the UV region with absorption peaks at 270 nm (Fig. 4a), which is hypsochromically shifted compared to those of the corresponding CPPs ([10]CPP: 340 nm; [12]CPP: 338 nm; [14]CPP: 338 nm, See Supplementary Fig. 9)^33,37^. While no obvious fluorescence was detected (*Φ* < 0.01) at room temperature, bright phosphorescence was observed at low temperature (≤150 K). As shown in Fig. 4a, the PFCPPs in ethanol grass exhibited phosphorescence with peak tops at 507 nm (PF[10]CPP), 500 nm (PF[12]CPP), and 492 nm (PF[14]CPP) at 77 K. The phosphorescence quantum yields (*Φ*) in ethanol grass at 77 K were 0.21 (PF[10]CPP), 0.62 (PF[12]CPP), and 0.38 (PF[14]CPP). The long lifetimes (*τ*) of 2.0 s (PF[10]CPP), 0.6 s (PF[12]CPP) and 0.7 s (PF[14]CPP) were also confirmed as the dispersed solid in poly(methyl methacrylate) (for details, see Supplementary Fig. 4 in SI). These photoluminescence properties clearly indicate that intersystem crossing occurs much more quickly compared to CPPs, which exhibit high-fluorescence quantum yields at room temperature ([10]CPP: 0.46–0.65; [12]CPP: 0.66–0.89, [14]CPP: 0.89)^33,37^.

Next, cyclic voltammograms were recorded (Fig. 4b). In acetonitrile, all PFCPPs showed a reduction potential (–1.23 V vs ferrocene(II)/ferrocenium(III)) higher than those of previously reported CPPs (e.g., [9]CPP: −2.45 V) and partially fluorinated CPPs (e.g., F_12_[9]CPP: −2.06 V), indicating that the perfluorination increases the electron affinity of CPPs^26–28^. Figure 4c shows the HOMO and LUMO of PF[10]CPP with their energies calculated at B3LYP/6-31G(d) level of theory (for details on PF[*n*]CPPs (*n* = 12, 14, 16), see Supplementary Fig. 9 in SI). While the shape and distribution of each frontier molecular orbital of PF[10]CPP are almost identical to that of [10]CPP, the HOMO–LUMO gap is wider (PF[10]CPP: 4.24 eV) than that of [10]CPP (3.54 eV)^33^, which is in line with the hypsochromic shift of the absorption spectra.

In summary, we have synthesized and isolated PF[*n*]CPPs (*n* = 10, 12, 14, 16), which represent highly strained perfluorocarbon molecules. The synthesis of these PFCPPs was accomplished in a one-pot fashion via deprotonation of octafluorobiphenyl, transmetallation to Ni(dnbpy)Br_2_, and oxidant-promoted reductive elimination. The high solubility of intermediates enhanced by the *n*-nonyl groups of dnbpy might be the key to the success of this concise synthesis. PF[*n*]CPPs (*n* = 10, 12, 14, 16) were identified by spectroscopic analysis (^19^F NMR and LDI-TOF MS, IR, Raman), and the solid-state structures of PF[10]CPP, PF[12]CPP, and PF[14]CPP were unambiguously determined by X-ray crystallography. In the crystal structure, the PFCPPs exhibit a tubular shape, and the ring cavities are connected one-dimensionally. The dihedral angles between pairs of benzene rings in the PFCPPs are larger than those of the corresponding CPPs due to the steric repulsion between fluorine atoms. Optical and electrochemical measurements revealed wide HOMO–LUMO gaps and high reduction potential for PF[*n*]CPPs (*n* = 10, 12, 14), which also show strong phosphorescence at low temperature. This noble metal-free one-pot synthesis of PFCPPs represents a huge breakthrough in fluorocarbon chemistry. Apart from the obvious interesting electronic features of strained PFCPPs, it should also be possible to create highly strained molecular nanocarbon materials by further converting the reactive C–F bonds of PFCPPs. PFCPPs are not only attractive as electron-deficient aromatic materials but are also potentially applicable to further transformations for the creation of highly strained and topologically unique molecular nanocarbon materials^38^.

## Methods

### Synthesis of PF[*n*]CPPs (*n* = 10, 12, 14, 16)

To a 200-mL two-necked round-bottomed flask containing a magnetic stirring bar and filled by argon gas were added 2,3,5,6,2′,3′,5′,6′-octafluorobiphenyl (**1**) (1.00 g, 3.35 mmol), Ni(dnbpy)Br_2_ (2.10 g, 3.35 mmol), and dry THF (67 mL). The 2.0 M solution of lithium diisopropylamide (LDA) in THF (6.75 mL) was added to the flask at −78 °C. After the reaction mixture was stirred for 30 min, volatile solvents were evaporated in vacuo. The flask was filled by argon gas, and 2,3-dichloro-5,6-dicyano-*p*-benzoquinone (DDQ, 3.81 g, 16.8 mmol) and degassed *m*-xylene (100 mL) were added to the flask. The reaction mixture was stirred at 130 °C for 5 h. After cooling the reaction mixture to room temperature, the reaction mixture was filtrated through Celite^®^ with chloroform (1.0 L), and the resulting filtrate was evaporated in vacuo. The crude product was purified by silica gel column chromatography (eluent: hexane/chloroform = 100:1 to 1:1) and then gel permeation chromatography (GPC; the crude solid (ca.100 mg) was dissolved in 120 mL chloroform, filtered with a Hydrophilic PTFE 0.45 µm Membrane filter (Millex-LCR 13 mm), and each 30 mL of resulting solution was injected to the GPC. Fractions were collected at the fourth cycle (see Supplementary Fig. 1).) to afford PF[*n*]CPPs (*n* = 10: 47.3 mg, 4.7%; *n* = 12: 22.3 mg, 2.2%; *n* = 14, 12.1 mg, 1.2%; *n* = 16: 6.7 mg, 0.7%) as a white solid.

## Supplementary information

Supplementary information

Description of Additional Supplementary Files

Supplementary Dataset 1

Supplementary Dataset 2

## Data Availability

Materials and methods, experimental procedures, photophysical studies, and NMR spectra are available in the Supplementary Information. Raw data corresponding to UV–Vis adsorption spectra (Fig. 4a) and Cyclic voltammograms (Fig. 4b) can be found in Supplementary Data File 1 and Supplementary Data 2, respectively. Crystallographic data for the structures reported in this article have been deposited at the Cambridge Crystallographic Data Centre under deposition numbers CCDC 2057897 (PF[10]CPP), 2057898 (PF[12]CPP), and 2133188 (PF[14]CPP). Copies of the data can be obtained free of charge via https://www.ccdc.cam.ac.uk/structures/.
