# Supplementary material for: Perfluorocycloparaphenylenes
Source: Nat Commun. 2022 Jun 28;13:3713. doi: 10.1038/s41467-022-31530-x (PMC9240036; doi:10.1038/s41467-022-31530-x)
Supplement: Supplementary file 1 — Supplementary information [file 41467_2022_31530_MOESM1_ESM.pdf]

Supplementary Information

Perfluorocycloparaphenylenes

Hiroki Shudo[1], Motonobu Kuwayama[2,3], Masafumi Shimasaki[4], Taishi Nishihara[4], Youhei Takeda[5], Nobuhiko Mitoma[6], Takuya Kuwabara[1,2,3], Akiko Yagi[1,3], Yasutomo Segawa[1,2,7,8,*] and Kenichiro Itami[1,2,3,*]

[1] *Graduate School of Science, Nagoya University, Nagoya 464-8602, Japan*

[2] *JST, ERATO, Itami Molecular Nanocarbon Project, Nagoya University, Nagoya 464-8602, Japan*

[3] *Institute of Transformative Bio-Molecules (WPI-ITbM) Nagoya University, Nagoya 464-8602, Japan*

[4] *Institute of Advanced Energy, Kyoto University, Kyoto 611-0011, Japan*

[5] *Department of Applied Chemistry, Graduate School of Engineering, Osaka University, Yamadaoka 2-1, Suita, Osaka 565-0871, Japan*

[6] *RIKEN Center for Emergent Matter Science, Wako 351-0198, Japan*

[7] *Institute for Molecular Science, Myodaiji, Okazaki 444-8787, Japan*

[8] *Department of Structural Molecular Science, SOKENDAI (The Graduate University for Advanced Studies), Myodaiji, Okazaki 444-8787, Japan*

*E-mail: segawa@ims.ac.jp (Y.S.), itami@chem.nagoya-u.ac.jp (K.I.)

Table of Contents

Supplementary Methods S2–S6
Supplementary Figures and Tables S7–S6
1. Synthesis and purification S7–S9
2. X-ray crystallography S10–S12
3. IR and Raman spectroscopy S13–S14
4. Photophysical measurement S15–S18
5. Electrochemical measurement S19
6. Supramolecular experiment S20
7. Computational study S21–S26
8. NMR spectra and HRMS of PFCPPs S27–S34
Supplementary references S35

Supplementary Methods

1. Materials and methods

Materials: Unless otherwise noted, all reactants or reagents including dry solvents were obtained from commercial suppliers and used as received. Unless otherwise noted, all reactions were performed with dry solvents under an atmosphere of argon in dried glassware using standard vacuum-line techniques. All work-up and purification procedures were carried out with reagent-grade solvents in air.

Purification: Analytical thin-layer chromatography (TLC) was performed using Wako silica gel 70 F_{254} coated plates (0.25 mm); detection with UV light. Flash column chromatography was performed with E. Kanto silica gel 60 N (spherical, neutral, 40–100 μm). The developed chromatogram was analyzed by a UV lamp (254 nm). Recycling preparative gel permeation chromatography (GPC) was performed with a JAI LC-9260II NEXT instrument equipped with JAIGEL-2HR-40 columns (40 mm I.D. × 600 mm × 2) using chloroform as an eluent.

NMR and MS: The high-resolution mass spectra (HRMS) were obtained from a JEOL JMS-S3000 SpiralTOF (LDI-TOF MS). Nuclear magnetic resonance (NMR) spectra were recorded on a JEOL JNM-ECA-600 (^{19}F 565 MHz, ^{13}C{^{19}F} 150 MHz) spectrometer. Chemical shifts for ^{19}F NMR are expressed in parts per million (ppm) relative to hexafluorobenzene (C_6F_6: δ −164.9 ppm). Chemical shifts for ^{13}C{^{19}F} NMR are expressed in parts per million (ppm) relative to $CDCl_3$ (δ 77.16 ppm). Data are reported as follows: chemical shift, multiplicity (s = singlet), and integration. For the NMR and MS spectra of PFCPPs, see Supplementary Figs. 16–31.

X-ray crystallography: Details of the crystal data and a summary of the intensity data collection parameters for **PF[*n*]CPP** (*n* = 10, 12, 14) are listed in Supplementary Table 1, and ORTEP drawings are shown in Supplementary Figs. 4–6. A suitable crystal was mounted with mineral oil on a MiTeGen MicroMounts and transferred to the goniometer of the kappa goniometer of a RIGAKU XtaLAB Synergy-S system with 1.2 kW MicroMax-007HF microfocus rotating anode (Graphite-monochromated Mo Kα radiation (λ = 0.71073 Å)) and PILATUS200K hybrid photon-counting detector. Cell parameters were determined and refined, and raw frame data were integrated using CrysAlis^{Pro} (Agilent Technologies, 2010). The structures were solved by direct methods with SHELXT[1] and refined by full-matrix least-squares techniques against F^2 (SHELXL-2018/3)[2] by using Olex2 software package[3]. The intensities were corrected for Lorentz and polarization effects. The non-hydrogen atoms were refined anisotropically. Hydrogen atoms were placed using AFIX instructions. CCDC 2057897, 2057898 and 2133188 contains the supplementary crystallographic data for this paper. These data can be obtained free of charge from The Cambridge Crystallographic Data Centre via www.ccdc.cam.ac.uk/data_request/cif.

IR and Raman: The IR spectra were recorded on JASCO FT/IR-6100 (KBr). The Raman spectra were measured using a confocal Raman microscope (inVia Reflex, Renishaw) equipped

with a semiconductor laser operated at 488 nm. The Raman signal was detected by thermoelectrically-cooled charge-coupled device (CCD). A 100x, 0.85 NA objective lens was used to focus the laser light onto the samples. Measurements were carried out at rt and atmospheric conditions. For the IR and Raman spectra of PFCPPs, see Supplementary Figs. 7 and 8.

Photophysical measurements: UV–Vis absorption spectra were recorded on a Shimadzu UV-3600 spectrometer with a resolution of 0.5 nm. Dilute solutions in spectral grade dichloromethane in a 1 cm square quartz cell were used for measurements. Absolute quantum yields (Φ) were determined with a Hamamatsu Photonics C11347-01 Absolute quantum yield spectrometer (Quantaurus-QY) equipped with an integrating sphere using a liquid N_2 Dewar condenser. For temperature-dependent phosphorescence measurements, the excitation light source was an ultraviolet lamp with the wavelength of 254 nm together with a bandpass filter (U-330). The phosphorescence spectra and decay were recorded with a monochromator attached to a thermoelectrically cooled charged-coupled device (CCD) camera (Princeton Instruments, ProEM). To omit short-lifetime emission, spectra were recorded with the delay time of 0.1 s after the UV lamp was turned off. The sample was prepared by drop-casting a chloroform solution of PMMA (poly(methyl methacrylate)) onto a silicon substrate. After air-drying of the solvent, the sample was set in the cryostat (JANIS, CCS-XG-M). For the absorption and photoluminescence spectra, see Supplementary Figs. 9–12.

Electrochemical measurements: Cyclic voltammetry (CV) was performed on a BAS ALS620A electrochemical analyzer. The CV cell consisted of a glassy carbon electrode, a Pt wire counter electrode, and a titanium reference electrode. The measurements were carried out under a nitrogen atmosphere using a acetonitrile solution of a sample with a concentration of 1.0 mM and 0.1 M tetrabutylammonium hexafluorophosphate (n-Bu_4NPF_6) as a supporting electrolyte. The redox potentials were calibrated with ferrocene as an internal standard. For the CV spectra, see Supplementary Figs. 13.

Supramolecular experiments: the $CDCl_3$ solution PF[n]CPPs (n = 10, 90 μM; n = 12, 97 μM) with hexafluorobenzene as an internal standard was prepared and ^{19}F NMR spectra were recorded. To the solution, 30 μL of the $CDCl_3$ solution of fullerene C_{60} ((n = 10, 9 equiv; n = 12, 13 equiv) was added and ^{19}F NMR spectra were recorded. As shown in Supplementary Fig. 14, broadening of the signal of PF[10]CPP was observed while PF[12]CPP showed no change, implying the existence of the interaction between PF[10]CPP and C_{60} in solution.

Computational study: The Gaussian 16 program[6] running on a NEC LX 110Rh system was used for optimization (B3LYP/6-31G(d)[7,8]. Structures were optimized without any symmetry assumptions. Structures were optimized without any symmetry assumptions. Zero-point energy, enthalpy, and Gibbs free energy at 298.15 K and 1 atm were estimated from the gas-phase studies.

Harmonic vibration frequency calculation at the same level was performed to verify all stationary points as local minima (with no imaginary frequency). For the detail of frontier molecular orbitals and strain energy of PFCPPs, see Supplementary Fig. 15. Energies and Cartesian coordinates are listed in Supplementary Tables 2 and 3.

2. Synthesis of PF[*n*]CPPs (*n* = 10, 12, 14, 16)

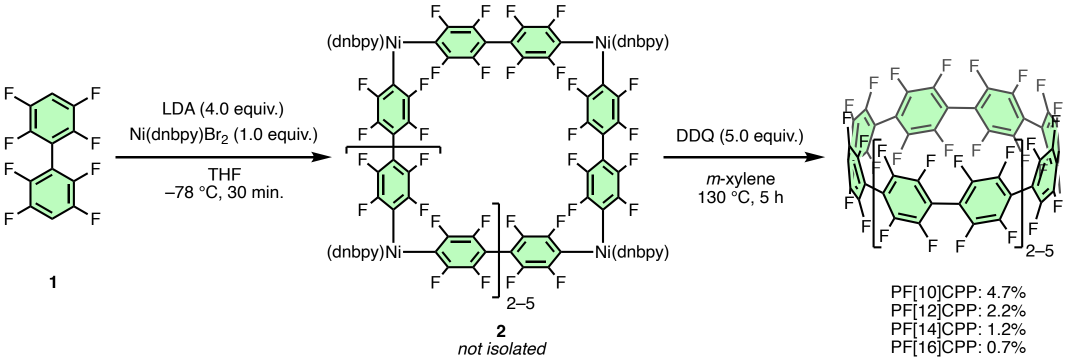

To a 2-L two-necked round-bottom flask containing a magnetic stirring bar were added 4,4'-dinonyl-2,2'-bipyridyl (24.5 g, 60.0 mmol), $NiBr_2$ (13.3 g, 60.6 mmol), and dry THF (1.0 L). The reaction mixture was stirred at 80 °C for 24 h. After cooling the reaction mixture to room temperature, the suspension was filtrated and the resulting filtrate was evaporated *in vacuo* to obtain Ni(dnbpy)Br_2 as a green solid (37.6 g), which was used without further purification.

To a 200-mL two-necked round-bottomed flask containing a magnetic stirring bar and filled by argon gas were added 2,3,5,6,2',3',5',6'-octafluorobiphenyl (**1**) (1.00 g, 3.35 mmol), Ni(dnbpy)Br_2 (2.10 g, 3.35 mmol), and dry THF (67 mL). The 2.0 M solution of lithium diisopropylamide (LDA) in THF (6.75 mL) was added to the flask at –78 °C. After the reaction mixture was stirred for 30 min, volatile solvents were evaporated *in vacuo*. The flask was filled by argon gas, and 2,3-dichloro-5,6-dicyano-*p*-benzoquinone (DDQ, 3.81 g, 16.8 mmol) and degassed *m*-xylene (100 mL) were added to the flask. The reaction mixture was stirred at 130 °C for 5 h. After cooling the reaction mixture to room temperature, the reaction mixture was filtrated through Celite® with chloroform (1.0 L), and the resulting filtrate was evaporated *in vacuo*. The crude product was purified by silica gel column chromatography (eluent: hexane/chloroform = 100:1 to 1:1) and then GPC (The crude solid (ca.100 mg) was dissolved in 120 mL chloroform, filtered with a Hydrophilic PTFE 0.45 µm Membrane filter (Millex-LCR 13 mm), and each 30 mL of resulting solution was injected to the GPC. Fractions were collected at the fourth cycle (see Supplementary Fig. 1).) to afford PF[*n*]CPPs (*n* = 10: 47.3 mg, 4.7%; *n* = 12: 22.3 mg, 2.2%; *n* = 14, 12.1 mg, 1.2%; *n* = 16: 6.7 mg, 0.7%) as a white solid.

PF[10]CPP: ^{19}F NMR (600 MHz) δ –138.3 (s, 40F); $^{13}C\{^{19}F\}$ NMR (150 MHz) δ 110.5 (s), 145.2 (s); HRMS (LDI-TOF MS) *m/z* calcd for $C_{60}F_{40}$ $[M]^-$: 1479.9367, found: 1479.9344.

PF[12]CPP: ^{19}F NMR (600 MHz) δ –138.5 (s, 48F); $^{13}C\{^{19}F\}$ NMR (150 MHz) δ 110.1 (s), 145.0 (s); HRMS (LDI-TOF MS) *m/z* calcd for $C_{72}F_{48}$ $[M]^-$: 1775.9239, found: 1775.9248.

PF[14]CPP: ^{19}F NMR (600 MHz) δ –138.6 (s, 56F); $^{13}C\{^{19}F\}$ NMR (150 MHz, 50 °C) δ 110.0 (s), 145.0 (s); HRMS (LDI-TOF MS) *m/z* calcd for $C_{84}F_{56}$ $[M]^-$: 2071.9111, found: 2071.9110.

PF[16]CPP: ^{19}F NMR (600 MHz) δ –138.8 (s, 64F); $^{13}C\{^{19}F\}$ NMR (150 MHz) δ 109.8 (s), 144.8 (s); HRMS (LDI-TOF MS) *m/z* calcd for $C_{96}F_{64}$ $[M]^{-}$: 2367.8984, found: 2367.8978.

Supplementary Figures and Tables

1. Synthesis and purification

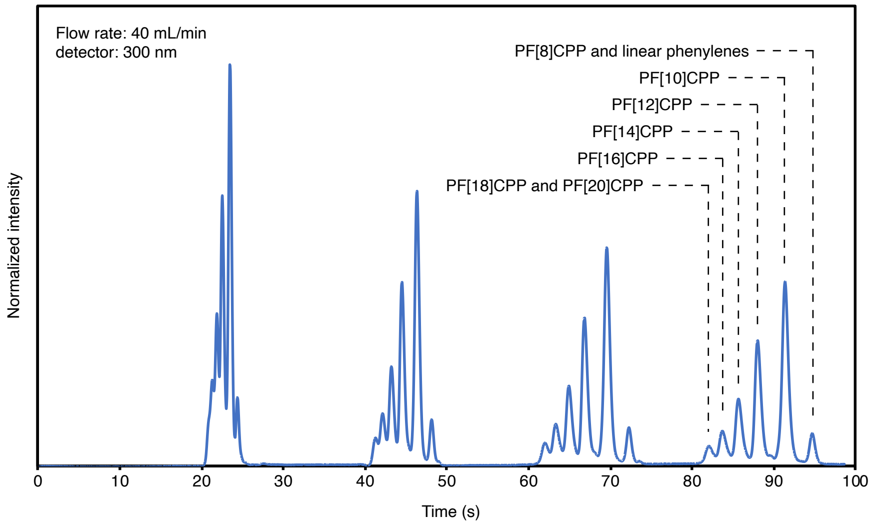

Supplementary Fig. 1. The chromatogram of recycling preparative GPC of the crude mixture containing PFCPPs.

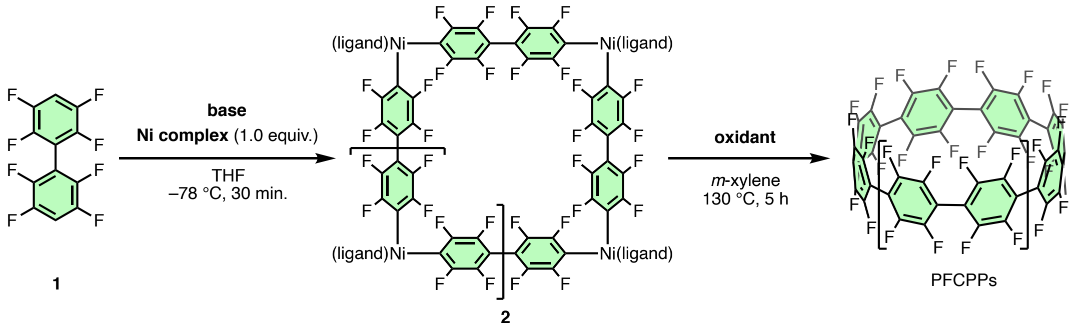

entry	base	Ni complex	oxidant	yield
1	LDA<br>(4 equiv.)	Ni(dnbpy)Br_2	DDQ<br>(5 equiv.)	n = 10 : 4.7%<br>n = 12 : 2.2%<br>n = 14 : 1.2%<br>n = 16 : 0.7%
2	LDA<br>(4 equiv.)	Ni(dnbpy)Br_2	TCNQ<br>(5 equiv.)	n = 10 : 3%<br>n = 12 : 2%<br>n = 14 : 1%<br>n = 16 : 0.6%
3	LiHMDS<br>(4 equiv.)	Ni(dnbpy)Br_2	TCNQ<br>(5 equiv.)	n = 10 : 1%<br>n = 12 : 2%<br>n = 14 : 1%<br>n = 16 : 0.8%
4	NaHMDS<br>(4 equiv.)	Ni(dnbpy)Br_2	TCNQ<br>(5 equiv.)	n.d.
5	TMPMgCl•LiCl<br>(4 equiv.)	Ni(dnbpy)Br_2	TCNQ<br>(5 equiv.)	n.d.
6	LDA<br>(4 equiv.)	Ni(bpy)Br_2	DDQ<br>(5 equiv.)	n.d.
7	LDA<br>(4 equiv.)	Ni(dtbpy)Br_2	DDQ<br>(5 equiv.)	trace
8	LDA<br>(4 equiv.)	Ni(dppf)Br_2	DDQ<br>(5 equiv.)	n.d.
9	LDA<br>(4 equiv.)	Ni(dppe)Br_2	DDQ<br>(5 equiv.)	n.d.

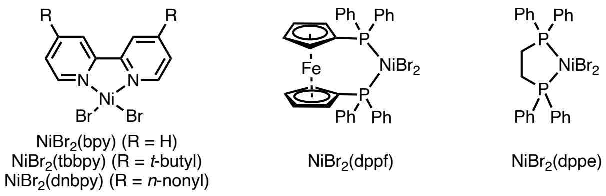

Supplementary Fig. 2. Screening of the reaction conditions.

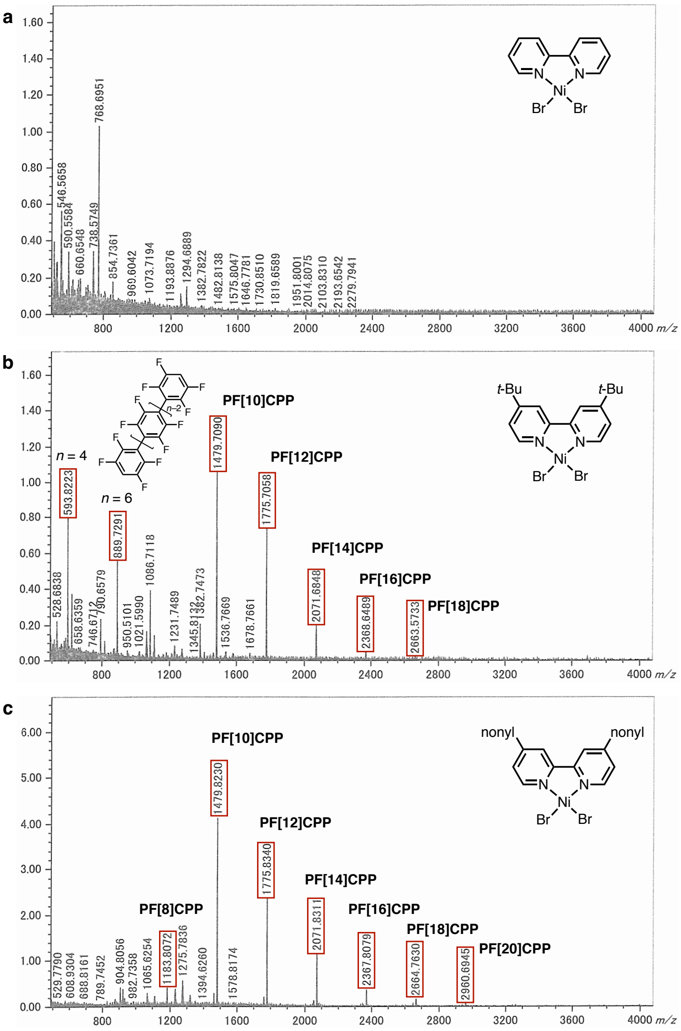

Supplementary Fig. 3. LDI-TOF mass spectra of crude mixture obtained in entries 6 (**a**), 7 (**b**), and 1 (**c**) in Fig. S1.

2. X-ray crystallography

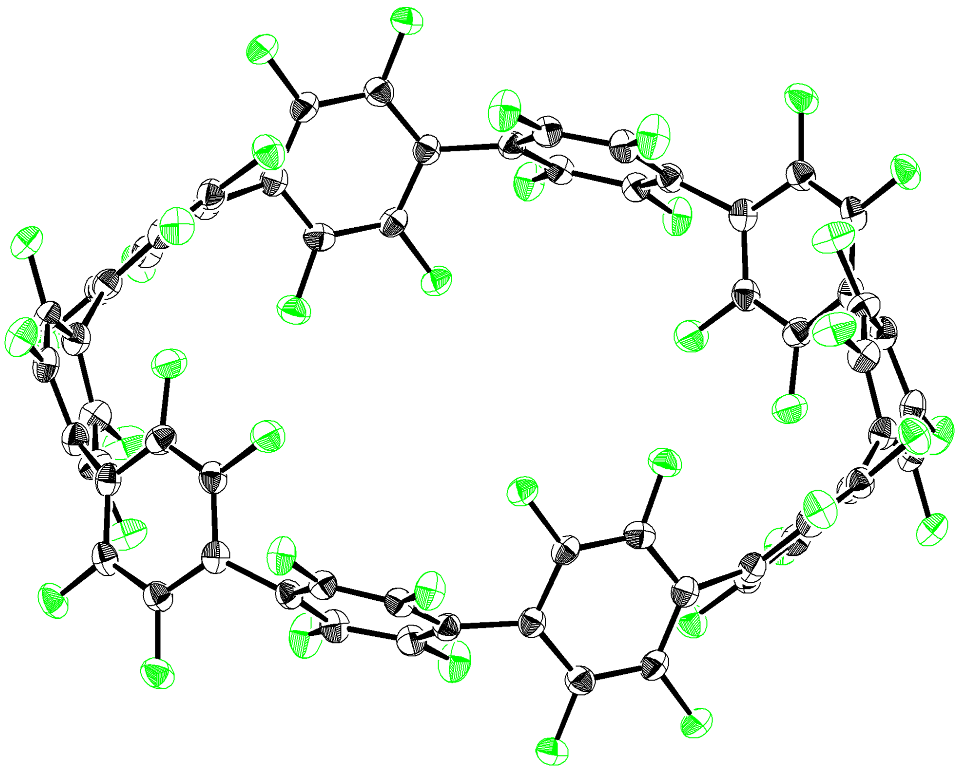

Supplementary Fig. 4. ORTEP (Oak Ridge Thermal-Ellipsoid Plot) of PF[10]CPP with 50% thermal probabilities. Solvent molecules are omitted for clarity.

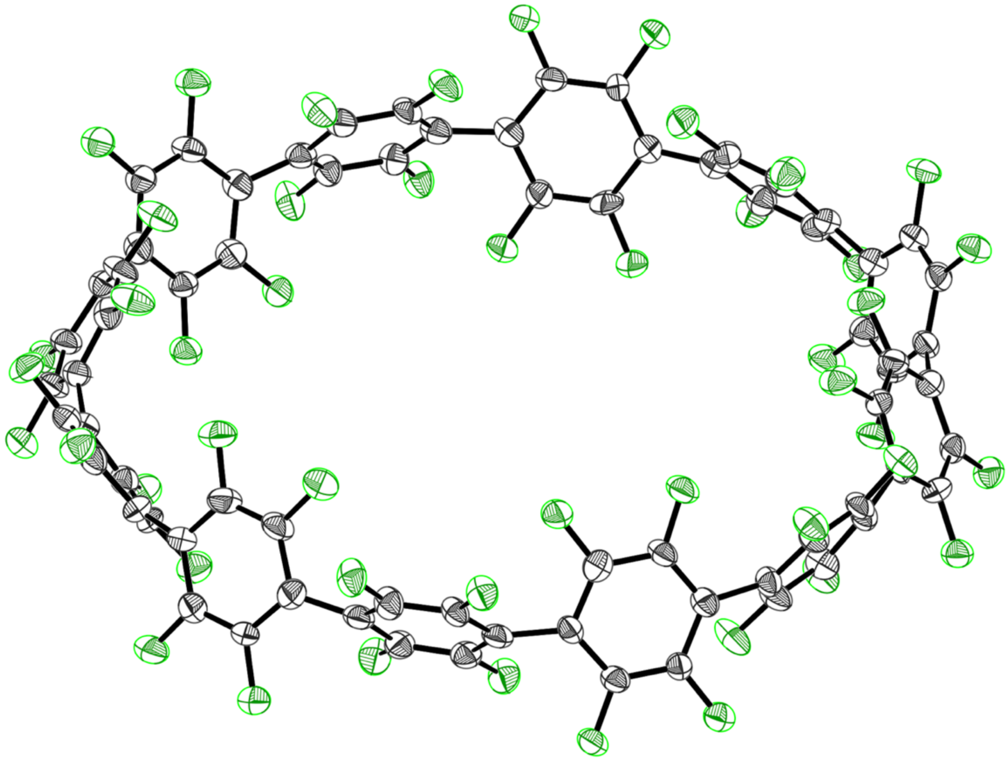

Supplementary Fig. 5. ORTEP of PF[12]CPP with 50% thermal probabilities. Solvent molecules

are omitted for clarity.

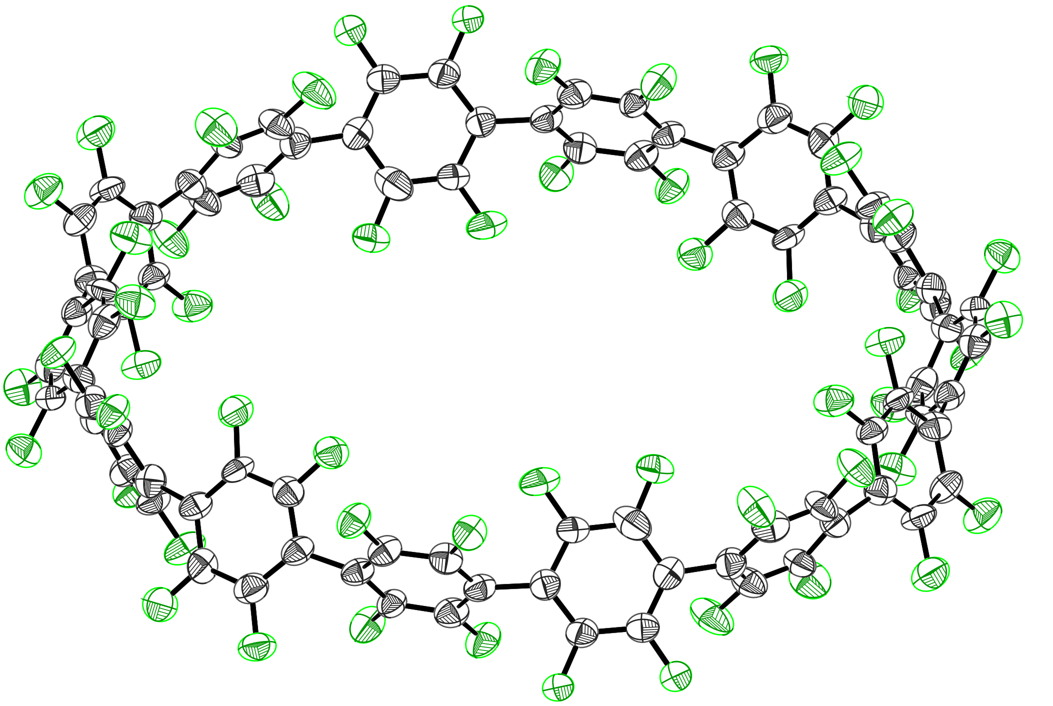

Supplementary Fig. 6. ORTEP of PF[14]CPP with 50% thermal probabilities. Solvent molecules are omitted for clarity.

Supplementary Table 1. Crystallographic data and structure refinement details of PF[*n*]CPPs (*n* = 10, 12, 14)

	PF[10]CPP	PF[12]CPP	PF[14]CPP
CCDC No.	2057897	2057898	2133188
formula	$C_{68}H_{16}F_{40}O_2$	$C_{96}F_{72}$	$C_{54.25}H_{28}Cl_3F_{28}$
fw	1624.81	2520.96	1318.11
T (K)	123(2)	123(2)	123(3)
λ (Å)	0.71073	0.71073	0.71073
cryst syst	monoclinic	triclinic	monoclinic
space group	$P2_1/c$	P-1	$P2_1/c$
a (Å)	8.4683(3)	13.4334(6)	7.5705(4)
b (Å)	16.4891(5)	20.5979(11)	37.065(2)
c (Å)	23.0124(7)	20.6206(12)	22.0824(13)
α (deg)	90	67.743(5)	90
β (deg)	97.639(3)	84.644(4)	98.144(5)
γ (deg)	90	76.374(4)	90
V (Å^3)	3184.81(18)	5131.9(5)	6133.8(6)
Z	2	2	4
D_{calc} (g·cm^{-3})	1.694	1.631	1.427
μ (mm^{-1})	0.183	0.188	0.269
F(000)	1600.0	2448.0	2626.0
cryst size (mm^3)	0.01 × 0.01 × 0.01	0.20 × 0.01 × 0.01	0.15 × 0.03 × 0.01
2θ range (deg)	4.342–50	3.772–50	3.726–55.912
reflns collected	45518	94735	47174
indep reflns/R_{int}	5589 / 0.0411	18056 / 0.1909	12420 / 0.2499
params	542	1945	894
GOF on F^2	1.033	1.043	1.081
R_1, wR_2 [I>$2\sigma(I)$]	0.0463, 0.1273	0.0954, 0.2127	0.1271, 0.3347
R_1, wR_2 (all data)	0.0621, 0.1384	0.2357, 0.2828	0.3105, 0.4187

3. IR and Raman spectroscopy

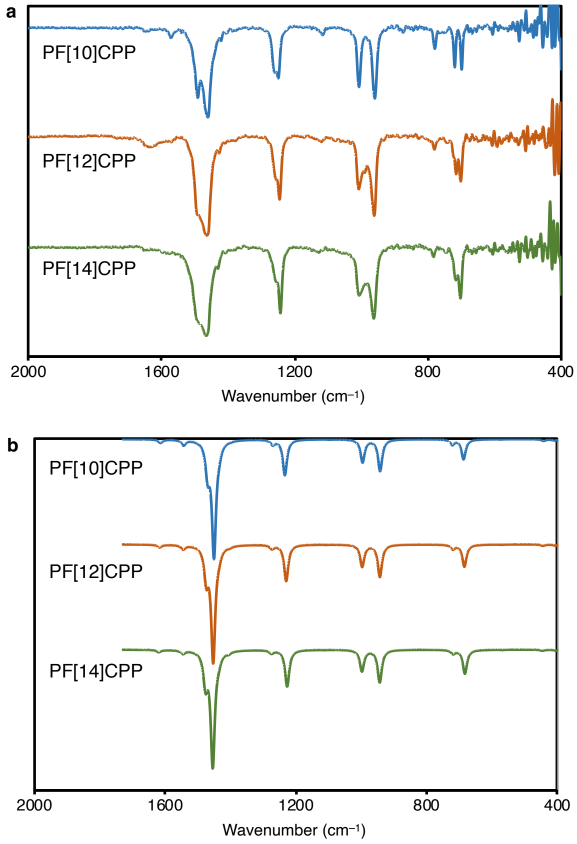

Supplementary Fig. 7. (**a**) IR spectra of PF[*n*]CPPs (*n* = 10, 12, 14). (**b**) Simulated IR spectra of PF[*n*]CPPs (*n* = 10, 12, 14) by B3LYP/6-31G(d) level of theory scaled with a factor of 0.9613.

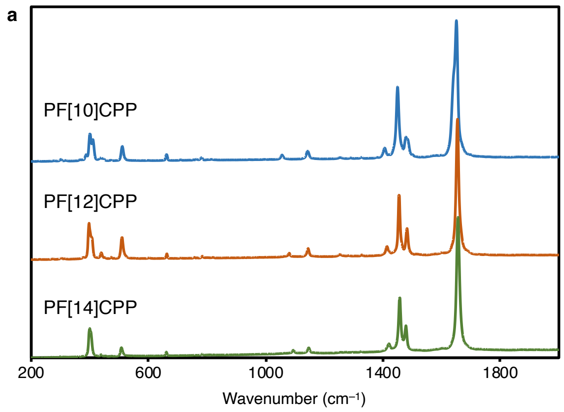

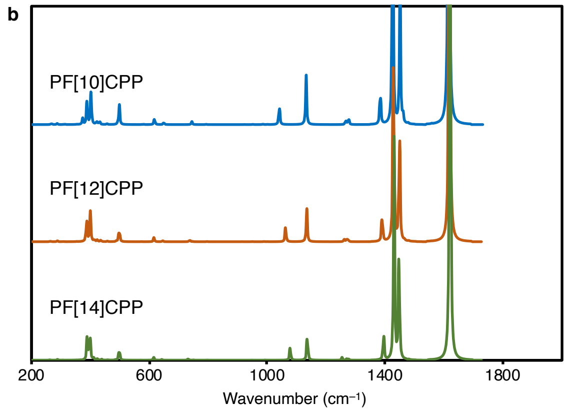

Supplementary Fig. 8. (**a**) Raman spectra of PF[*n*]CPPs (*n* = 10, 12, 14). (**b**) Simulated Raman spectra of PF[*n*]CPPs (*n* = 10, 12, 14) by B3LYP/6-31G(d) level of theory scaled with a factor of 0.9613.

4. Photophysical measurement

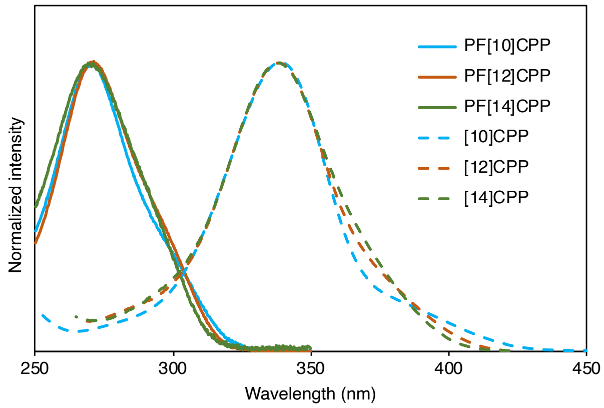

Supplementary Fig. 9. UV–Vis absorption spectra of the diluted dichloromethane solution of PFCPPs and CPPs[4,5].

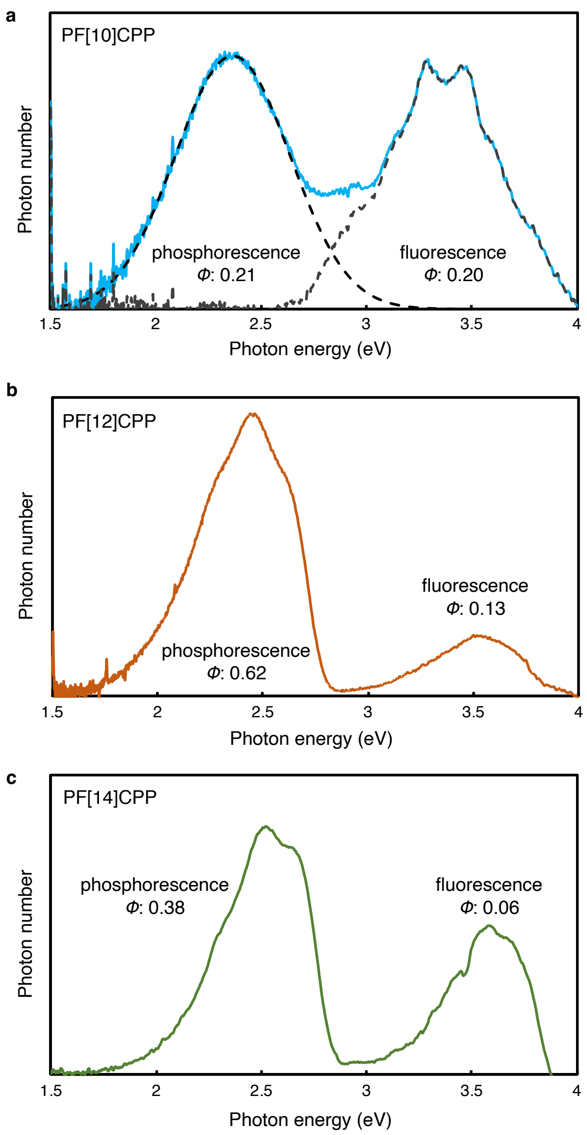

Supplementary Fig. 10. Phosphorescence and fluorescence spectra of PF[n]CPPs (n = 10 (**a**), 12 (**b**), 14 (**c**)) in ethanol grass at 77 K upon excitation at 270 nm. The total quantum yields (Φ) of PF[10]CPP, PF[12]CPP, and PF[14]CPP were 0.41, 0.75, and 0.44 respectively. For PF[10]CPP, phosphorescence and fluorescence components were divided by curve fitting of phosphorescence to a Gaussian function as shown with dashed lines. For PF[12]CPP and PF[14]CPP, phosphorescence and fluorescence components were divided at 2.85 and 2.88 eV, respectively.

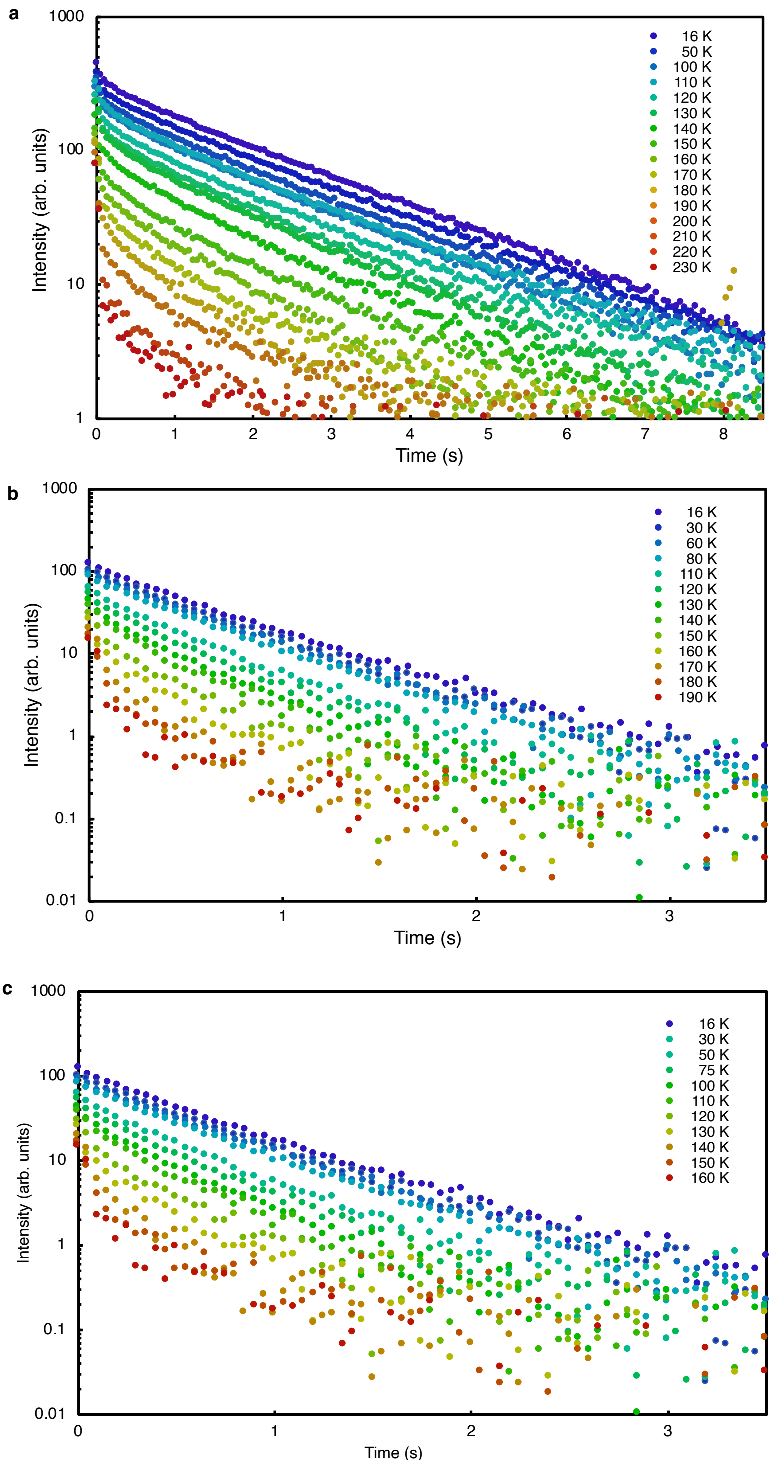

Supplementary Fig. 11. Plots of phosphorescence intensity versus relaxation time of PFCPPs dispersed in PMMA matrix upon excitation at 254 nm. (**a**) Phosphorescence intensity of PF[10]CPP from 16 K to 230 K (τ = 2.0 s). (**b**) Phosphorescence intensity of PF[12]CPP from 16 K to 190 K (τ = 0.6 s). (**c**) Phosphorescence intensity of PF[14]CPP from 16 K to 160 K (τ = 0.7 s).

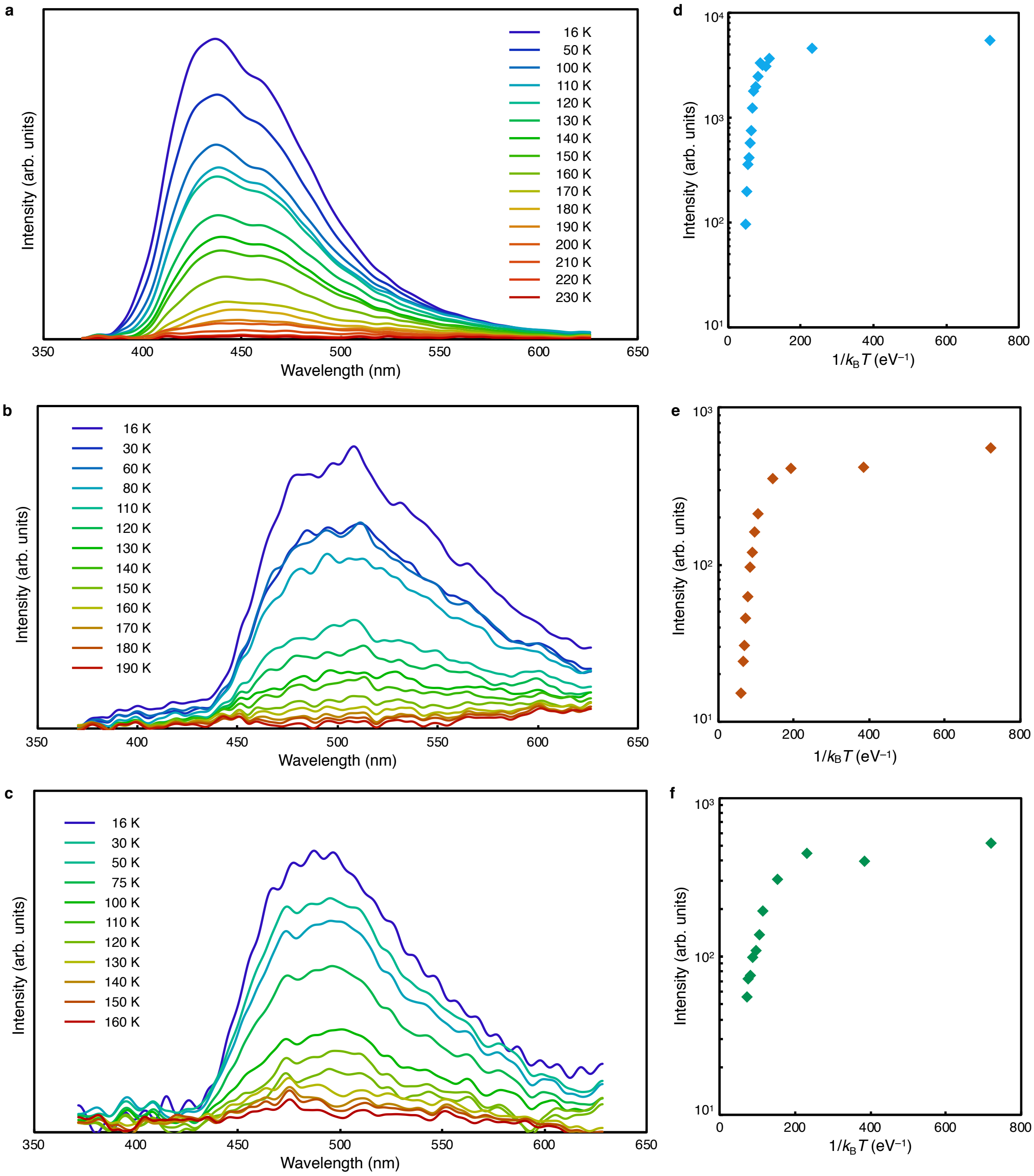

Supplementary Fig. 12. (**a**–**c**) Temperature-dependent phosphorescence spectra of PF[10]CPP (**a**), PF[12]CPP (**b**), and PF[14]CPP (**c**) dispersed in PMMA matrix at 16 K upon excitation at 254 nm. Delay time: 0.1 s. (**d**–**f**) Integrated phosphorescence intensity from 400 nm to 600 nm versus inverse temperature of PF[10]CPP (**d**), PF[12]CPP (**e**), and PF[14]CPP (**f**) dispersed in PMMA matrix.

5. Electrochemical measurement

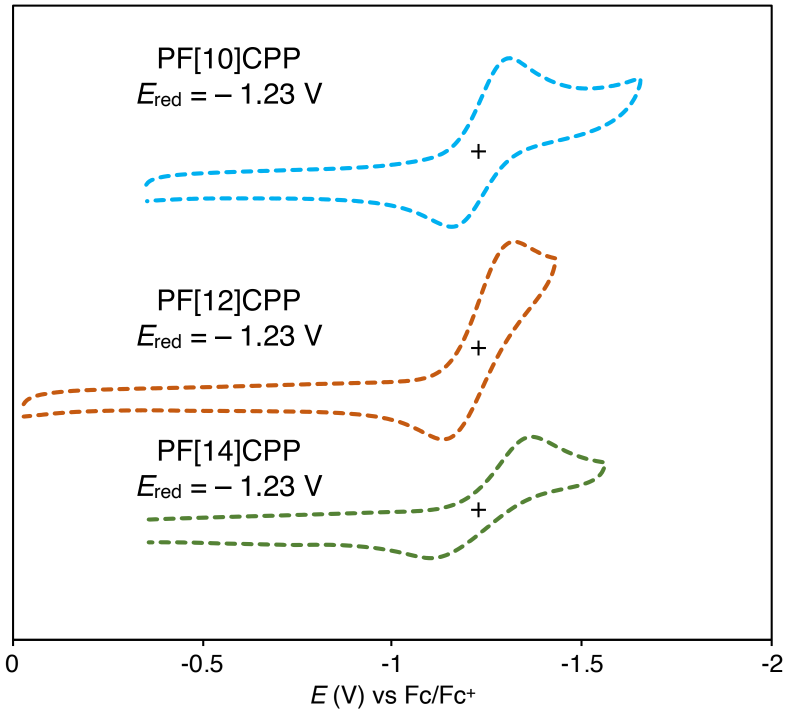

Supplementary Fig. 13. Cyclic voltammograms of PFCPPs in acetonitrile, measured with *n*-Bu_4NPF_6 (0.1 M) as a supporting electrolyte at a scan rate of 100 mV s^{-1}.

6. Supramolecular experiment

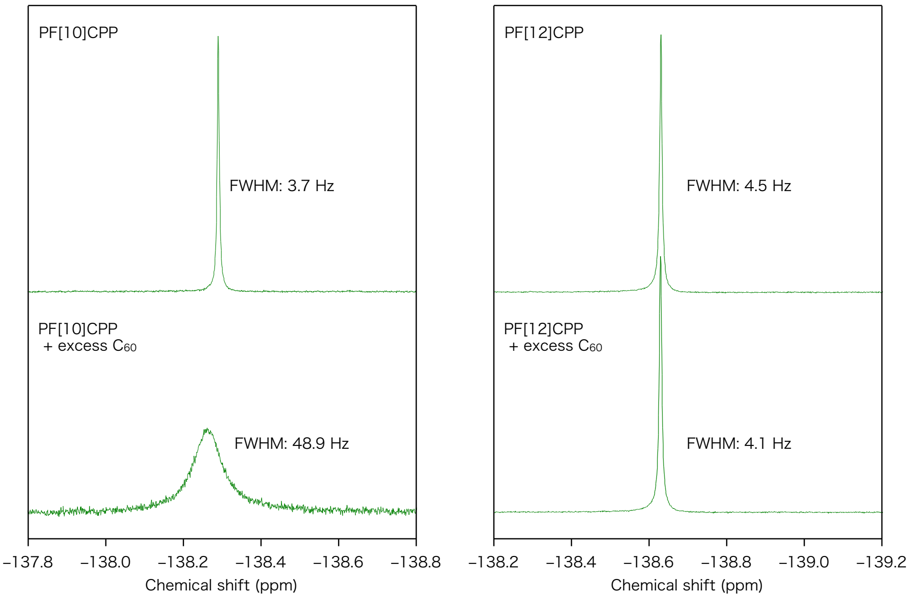

Supplementary Fig. 14. The ^{19}F NMR spectra of $CDCl_3$ solution PF[*n*]CPPs (*n* = 10, 12) with or without C_{60} solution. Hexafluorobenzene was used as the internal reference of chemical shift (0.0 ppm). FWHM: full width at half maximun. The full spectra are shown in Supplementary Figs. 28–31.

7. Computational study

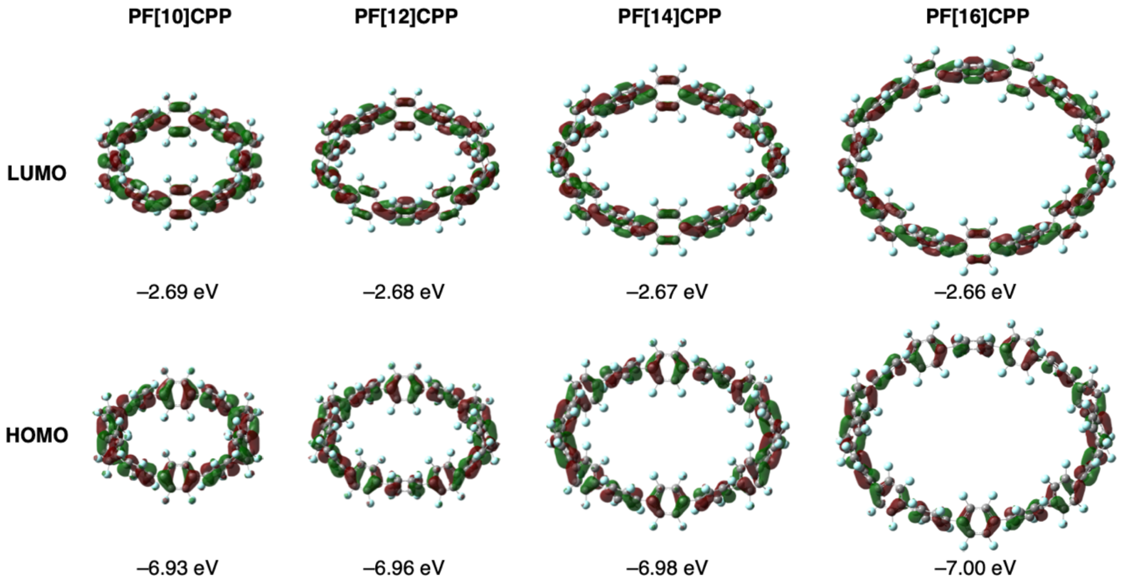

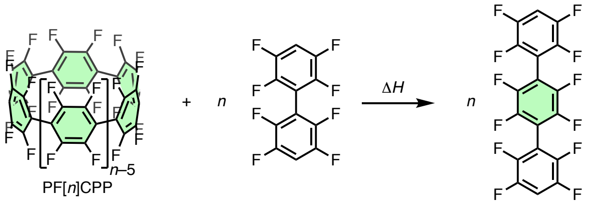

n	strain energy (kcal·mol^{-1})
6	101.8
8	75.8
10	60.2
12	49.9
14	42.6
16	37.2

Supplementary Fig. 15. (top) Frontier molecular orbitals of PF[*n*]CPPs (*n* = 10, 12, 14, 16). Isovalue: 0.02. (bottom) Hypothetical homodesmotic reactions[9] for the estimation of the strain energies of PF[*n*]CPPs.

Supplementary Table 2. Uncorrected and thermal-corrected (298 K) energies of stationary points (Hartree).[a]

	E	*E + ZPE*	*H*	*G*
**PF[6]CPP**	-3767.634276	-3767.348167	-3767.299886	-3767.423218
**PF[8]CPP**	-5023.609609	-5023.227283	-5023.162008	-5023.322128
**PF[10]CPP**	-6279.568060	-6279.089812	-6279.007463	-6279.205153
**PF[12]CPP**	-7535.517993	-7534.944004	-7534.844563	-7535.080247
**PF[14]CPP**	-8791.463128	-8790.793397	-8790.676889	-8790.950579
**PF[16]CPP**	-10047.405218	-10046.639816	-10046.506254	-10046.820039
**Perfluorobiphenyl**	-1455.564966	-1455.465649	-1455.446481	-1455.511959
**Perfluoro-*p*-terphenyl**	-2083.531634	-2083.384475	-2083.356826	-2083.441773

a) E: electronic energy; ZPE: zero-point energy; H ($= E + ZPE + E_{vib} + E_{rot} + E_{trans} + RT$): sum of electronic and thermal enthalpies; G ($= H - TS$): sum of electronic and thermal free energies.

Supplementary Table 3. Cartesian coordinates of optimized structures.

PF[6]CPP

C	0.694090	3.536400	1.251218	C	3.409657	-1.167100	1.251218	C	-3.409657	-1.167100	1.251218
C	-0.694090	3.536400	1.251218	C	4.341579	-1.700055	-0.865695	C	-2.715567	-2.369299	1.251218
C	-0.698499	4.609945	-0.865695	C	3.643080	-2.909890	-0.865695	C	-3.643080	-2.909890	-0.865695
C	0.698499	4.609945	-0.865695	F	3.777325	-3.712709	-1.927105	C	-4.341579	-1.700055	-0.865695
F	1.326638	5.127613	-1.927105	F	5.103962	-1.414905	-1.927105	F	-5.103962	-1.414905	-1.927105
F	-1.326638	5.127613	-1.927105	C	4.140094	-0.733847	0.131270	F	-3.777325	-3.712709	-1.927105
C	-1.434517	3.952350	0.131270	C	-0.698499	-4.609945	0.865695	C	-2.705577	-3.218504	0.131270
C	4.341579	1.700055	0.865695	C	0.698499	-4.609945	0.865695	C	-3.643080	2.909890	0.865695
C	3.643080	2.909890	0.865695	C	1.434517	-3.952350	-0.131270	C	-4.341579	1.700055	0.865695
C	2.705577	3.218504	-0.131270	C	0.694090	-3.536400	-1.251218	C	-4.140094	0.733847	-0.131270
C	2.715567	2.369299	-1.251218	C	-0.694090	-3.536400	-1.251218	C	-3.409657	1.167100	-1.251218
C	3.409657	1.167100	-1.251218	C	-1.434517	-3.952350	-0.131270	C	-2.715567	2.369299	-1.251218
F	3.164457	0.329649	-2.267639	F	-1.296744	-2.905325	-2.267639	F	-1.867713	2.575676	-2.267639
F	1.867713	2.575676	-2.267639	F	1.296744	-2.905325	-2.267639	F	-3.164457	0.329649	-2.267639
C	1.434517	3.952350	0.131270	C	2.705577	-3.218504	0.131270	C	-4.140094	-0.733847	0.131270
F	5.103962	1.414905	1.927105	F	-1.326638	-5.127613	1.927105	F	-3.777325	3.712709	1.927105
F	3.777325	3.712709	1.927105	F	1.326638	-5.127613	1.927105	F	-5.103962	1.414905	1.927105
F	1.296744	2.905325	2.267639	F	1.867713	-2.575676	2.267639	F	-3.164457	-0.329649	2.267639
F	-1.296744	2.905325	2.267639	F	3.164457	-0.329649	2.267639	F	-1.867713	-2.575676	2.267639
C	2.715567	-2.369299	1.251218	C	4.140094	0.733847	-0.131270	C	-2.705577	3.218504	-0.131270

PF[8]CPP

F	3.665089	5.547999	1.981481	C	3.974866	2.993365	-1.202117	F	1.331419	-6.514637	-1.981481
F	5.547999	3.665089	1.981481	C	6.000649	0.697694	-0.921164	C	0.697694	-6.000649	-0.921164
F	5.547999	-3.665089	1.981481	C	6.000649	-0.697694	-0.921164	C	-0.697694	-6.000649	-0.921164
F	3.665089	-5.547999	1.981481	C	3.974866	-2.993365	-1.202117	F	-1.331419	-6.514637	-1.981481
F	-1.331419	6.514637	-1.981481	C	2.993365	-3.974866	-1.202117	C	-2.993365	-3.974866	-1.202117
F	1.331419	6.514637	-1.981481	F	-3.665089	5.547999	1.981481	C	-3.974866	-2.993365	-1.202117
F	6.514637	1.331419	-1.981481	F	-3.665089	-5.547999	1.981481	F	-6.514637	-1.331419	-1.981481
F	6.514637	-1.331419	-1.981481	F	-5.547999	-3.665089	1.981481	C	-6.000649	-0.697694	-0.921164
C	-1.431630	5.381169	0.097765	F	-5.547999	3.665089	1.981481	C	-6.000649	0.697694	-0.921164
C	-0.694027	4.927283	1.202117	C	-4.736444	3.749756	0.921164	F	-6.514637	1.331419	-1.981481
C	0.694027	4.927283	1.202117	C	-3.749756	4.736444	0.921164	F	-1.310363	4.310838	2.219253
C	1.431630	5.381169	0.097765	C	-2.792746	4.817376	-0.097765	F	1.310363	4.310838	2.219253
C	2.792746	4.817376	-0.097765	C	1.431630	-5.381169	0.097765	F	4.310838	1.310363	2.219253
C	3.749756	4.736444	0.921164	C	0.694027	-4.927283	1.202117	F	4.310838	-1.310363	2.219253
C	4.736444	3.749756	0.921164	C	-0.694027	-4.927283	1.202117	F	2.121656	3.974790	-2.219253
C	4.817376	2.792746	-0.097765	C	-1.431630	-5.381169	0.097765	F	3.974790	2.121656	-2.219253
C	5.381169	1.431630	0.097765	C	-2.792746	-4.817376	-0.097765	F	3.974790	-2.121656	-2.219253
C	4.927283	0.694027	1.202117	C	-3.749756	-4.736444	0.921164	F	2.121656	-3.974790	-2.219253

C	4.927283	-0.694027	1.202117	C	-4.736444	-3.749756	0.921164	F	1.310363	-4.310838	2.219253
C	5.381169	-1.431630	0.097765	C	-4.817376	-2.792746	-0.097765	F	-1.310363	-4.310838	2.219253
C	4.817376	-2.792746	-0.097765	C	-5.381169	-1.431630	0.097765	F	-4.310838	-1.310363	2.219253
C	4.736444	-3.749756	0.921164	C	-4.927283	-0.694027	1.202117	F	-4.310838	1.310363	2.219253
C	3.749756	-4.736444	0.921164	C	-4.927283	0.694027	1.202117	F	-3.974790	2.121656	-2.219253
C	2.792746	-4.817376	-0.097765	C	-5.381169	1.431630	0.097765	F	-2.121656	3.974790	-2.219253
C	-0.697694	6.000649	-0.921164	C	-4.817376	2.792746	-0.097765	F	-2.121656	-3.974790	-2.219253
C	0.697694	6.000649	-0.921164	C	-3.974866	2.993365	-1.202117	F	-3.974790	-2.121656	-2.219253
C	2.993365	3.974866	-1.202117	C	-2.993365	3.974866	-1.202117				

PF[10]CPP

C	4.905761	5.566301	0.952361	C	-3.149468	-5.515662	1.173976	F	-2.289483	5.392887	-2.193439
C	5.146014	4.650735	-0.078171	C	-4.272467	-4.699755	1.173976	F	-4.421452	3.843922	-2.193439
C	4.272467	4.699755	-1.173976	C	-0.697054	-7.386766	0.952361	F	-5.724224	5.609730	2.009998
C	3.149468	5.515662	-1.173976	C	-1.429579	-6.787274	-0.078171	F	-3.566288	7.177562	2.009998
C	2.832906	6.331306	-0.078171	C	-0.694051	-6.313475	-1.173976	F	-5.022091	3.017213	2.193439
C	3.777903	6.385737	0.952361	C	0.694051	-6.313475	-1.173976	F	-7.104053	3.710559	-2.009998
C	1.429579	6.787274	0.078171	C	1.429579	-6.787274	-0.078171	F	-7.928311	1.173752	-2.009998
C	0.697054	7.386766	-0.952361	C	0.697054	-7.386766	0.952361	F	-5.836430	0.510934	2.193439
C	-0.697054	7.386766	-0.952361	C	2.832906	-6.331306	0.078171	F	-5.836430	-0.510934	-2.193439
C	-1.429579	6.787274	0.078171	C	3.777903	-6.385737	-0.952361	F	-5.022091	-3.017213	-2.193439
C	-0.694051	6.313475	1.173976	C	4.905761	-5.566301	-0.952361	F	-7.104053	-3.710559	2.009998
C	0.694051	6.313475	1.173976	C	5.146014	-4.650735	0.078171	F	-7.928311	-1.173752	2.009998
C	-2.832906	6.331306	-0.078171	C	4.272467	-4.699755	1.173976	F	-5.724224	-5.609730	-2.009998
C	-3.149468	5.515662	-1.173976	C	3.149468	-5.515662	1.173976	F	-3.566288	-7.177562	-2.009998
C	-4.272467	4.699755	-1.173976	C	6.013317	-3.456994	-0.078171	F	-2.289483	-5.392887	2.193439
C	-5.146014	4.650735	-0.078171	C	5.789998	-2.611053	-1.173976	F	-4.421452	-3.843922	2.193439
C	-4.905761	5.566301	0.952361	C	6.218945	-1.290889	-1.173976	F	-1.333678	-7.902981	2.009998
C	-3.777903	6.385737	0.952361	C	6.896846	-0.737772	-0.078171	F	-1.317629	-5.708662	-2.193439
C	-5.789998	2.611053	1.173976	C	7.240633	-1.619698	0.952361	F	1.317629	-5.708662	-2.193439
C	-6.013317	3.456994	0.078171	C	6.809830	-2.945574	0.952361	F	1.333678	-7.902981	2.009998
C	-6.809830	2.945574	-0.952361	C	6.896846	0.737772	0.078171	F	3.566288	-7.177562	-2.009998
C	-7.240633	1.619698	-0.952361	C	7.240633	1.619698	-0.952361	F	5.724224	-5.609730	-2.009998
C	-6.896846	0.737772	0.078171	C	6.809830	2.945574	-0.952361	F	4.421452	-3.843922	2.193439
C	-6.218945	1.290889	1.173976	C	6.013317	3.456994	0.078171	F	2.289483	-5.392887	2.193439
C	-6.896846	-0.737772	-0.078171	C	5.789998	2.611053	1.173976	F	5.022091	-3.017213	-2.193439
C	-6.218945	-1.290889	-1.173976	C	6.218945	1.290889	1.173976	F	5.836430	-0.510934	-2.193439
C	-5.789998	-2.611053	-1.173976	F	5.724224	5.609730	2.009998	F	7.928311	-1.173752	2.009998
C	-6.013317	-3.456994	-0.078171	F	4.421452	3.843922	-2.193439	F	7.104053	-3.710559	2.009998
C	-6.809830	-2.945574	0.952361	F	2.289483	5.392887	-2.193439	F	7.928311	1.173752	-2.009998
C	-7.240633	-1.619698	0.952361	F	3.566288	7.177562	2.009998	F	7.104053	3.710559	-2.009998
C	-5.146014	-4.650735	0.078171	F	1.333678	7.902981	-2.009998	F	5.022091	3.017213	2.193439
C	-4.905761	-5.566301	-0.952361	F	-1.333678	7.902981	-2.009998	F	5.836430	0.510934	2.193439
C	-3.777903	-6.385737	-0.952361	F	-1.317629	5.708662	2.193439				
C	-2.832906	-6.331306	0.078171	F	1.317629	5.708662	2.193439				

PF[12]CPP

F	-3.489516	8.713298	2.028323	C	7.247522	4.988804	-0.972839	C	-4.450363	-6.319932	-1.155124
F	-1.321818	7.102236	2.176973	C	7.944192	3.782137	-0.972839	C	-5.329354	-6.373424	-0.064766
F	1.321818	7.102236	2.176973	C	7.802068	2.854871	0.064766	C	-6.373424	-5.329354	0.064766
F	3.489516	8.713298	2.028323	C	-7.802068	2.854871	0.064766	C	-6.319932	-4.450363	1.155124
F	5.801179	7.378659	2.028323	C	-7.944192	3.782137	-0.972839	C	-7.014094	-3.248040	1.155124
F	5.489807	4.695846	2.176973	C	-7.247522	4.988804	-0.972839	C	-7.802068	-2.854871	0.064766
F	6.811626	2.406390	2.176973	C	-6.373424	5.329354	0.064766	C	-8.184224	-1.428644	-0.064766
F	-6.811626	2.406390	2.176973	C	-5.329354	6.373424	-0.064766	C	-7.698403	-0.694162	-1.155124
F	-5.489807	4.695846	2.176973	C	-4.450363	6.319932	-1.155124	C	-7.698403	0.694162	-1.155124
F	-5.801179	7.378659	2.028323	C	-3.248040	7.014094	-1.155124	C	-8.184224	1.428644	-0.064766
F	-2.406390	6.811626	-2.176973	F	9.290696	1.334639	2.028323	F	7.102236	1.321818	-2.176973
F	-1.334639	9.290696	-2.028323	C	8.770941	0.696670	0.972839	F	7.102236	-1.321818	-2.176973
F	1.334639	9.290696	-2.028323	C	8.770941	-0.696670	0.972839	F	8.713298	-3.489516	-2.028323
F	2.406390	6.811626	-2.176973	F	9.290696	-1.334639	2.028323	F	7.378659	-5.801179	-2.028323
F	4.695846	5.489807	-2.176973	F	6.811626	-2.406390	2.176973	F	-2.406390	-6.811626	-2.176973
F	7.378659	5.801179	-2.028323	C	7.014094	-3.248040	1.155124	F	-4.695846	-5.489807	-2.176973
F	8.713298	3.489516	-2.028323	C	6.319932	-4.450363	1.155124	F	-5.489807	-4.695846	2.176973
F	-8.713298	3.489516	-2.028323	F	5.489807	-4.695846	2.176973	F	-6.811626	-2.406390	2.176973
F	-7.378659	5.801179	-2.028323	F	-3.489516	-8.713298	2.028323	F	-7.102236	-1.321818	-2.176973

F	-4.695846	5.489807	-2.176973
C	-0.694162	7.698403	1.155124
C	0.694162	7.698403	1.155124
C	3.782137	7.944192	0.972839
C	4.988804	7.247522	0.972839
C	6.319932	4.450363	1.155124
C	7.014094	3.248040	1.155124
C	-7.014094	3.248040	1.155124
C	-6.319932	4.450363	1.155124
C	-4.988804	7.247522	0.972839
C	-3.782137	7.944192	0.972839
C	-2.854871	7.802068	-0.064766
C	-1.428644	8.184224	0.064766
C	-0.696670	8.770941	-0.972839
C	0.696670	8.770941	-0.972839
C	1.428644	8.184224	0.064766
C	2.854871	7.802068	-0.064766
C	3.248040	7.014094	-1.155124
C	4.450363	6.319932	-1.155124
C	5.329354	6.373424	-0.064766
C	6.373424	5.329354	0.064766
C	-3.782137	-7.944192	0.972839
C	-4.988804	-7.247522	0.972839
F	-5.801179	-7.378659	2.028323
F	-7.378659	-5.801179	-2.028323
C	-7.247522	-4.988804	-0.972839
C	-7.944192	-3.782137	-0.972839
F	-8.713298	-3.489516	-2.028323
F	-9.290696	-1.334639	2.028323
C	-8.770941	-0.696670	0.972839
C	-8.770941	0.696670	0.972839
F	-9.290696	1.334639	2.028323
C	8.184224	1.428644	-0.064766
C	7.698403	0.694162	-1.155124
C	7.698403	-0.694162	-1.155124
C	8.184224	-1.428644	-0.064766
C	7.802068	-2.854871	0.064766
C	7.944192	-3.782137	-0.972839
C	7.247522	-4.988804	-0.972839
C	6.373424	-5.329354	0.064766
C	-2.854871	-7.802068	-0.064766
C	-3.248040	-7.014094	-1.155124
F	-7.102236	1.321818	-2.176973
F	5.801179	-7.378659	2.028323
C	4.988804	-7.247522	0.972839
C	3.782137	-7.944192	0.972839
F	3.489516	-8.713298	2.028323
F	1.321818	-7.102236	2.176973
C	0.694162	-7.698403	1.155124
C	-0.694162	-7.698403	1.155124
F	-1.321818	-7.102236	2.176973
C	5.329354	-6.373424	-0.064766
C	4.450363	-6.319932	-1.155124
C	3.248040	-7.014094	-1.155124
C	2.854871	-7.802068	-0.064766
C	1.428644	-8.184224	0.064766
C	0.696670	-8.770941	-0.972839
C	-0.696670	-8.770941	-0.972839
C	-1.428644	-8.184224	0.064766
F	4.695846	-5.489807	-2.176973
F	2.406390	-6.811626	-2.176973
F	1.334639	-9.290696	-2.028323
F	-1.334639	-9.290696	-2.028323

PF[14]CPP

F	-3.428302	10.197575	2.040508
F	-1.324140	8.492139	2.166777
F	1.324140	8.492139	2.166777
F	10.110297	3.677730	2.040508
F	10.704770	1.073173	2.040508
F	8.573872	-0.598738	2.166777
F	7.984574	-3.180620	2.166777
F	-7.465009	4.259508	2.166777
F	-5.813834	6.330016	2.166777
F	-5.835274	9.038439	2.040508
F	-2.491592	8.225675	-2.166777
F	-1.335769	10.675182	-2.040508
F	1.335769	10.675182	-2.040508
F	7.984574	3.180620	-2.166777
F	8.573872	0.598738	-2.166777
F	10.704770	-1.073173	-2.040508
F	10.110297	-3.677730	-2.040508
F	-9.179032	5.611521	-2.040508
F	-7.513356	7.700213	-2.040508
F	-4.877610	7.076630	-2.166777
C	-0.694296	9.081638	1.142566
C	0.694296	9.081638	1.142566
C	9.743704	2.938221	0.987168
C	10.053620	1.580390	0.987168
C	9.008438	-1.343966	1.142566
C	8.699447	-2.697743	1.142566
C	-7.533197	5.119486	1.142566
C	-6.667424	6.205131	1.142566
C	-5.032731	8.845594	0.987168
C	-3.777907	9.449885	0.987168
C	-2.867819	9.245721	-0.055692
C	-1.427753	9.574406	0.055692
C	-0.696375	10.153225	-0.987168
C	0.696375	10.153225	-0.987168
C	1.427753	9.574406	0.055692
C	9.016651	3.522462	-0.055692
C	8.699447	2.697743	-1.142566
C	9.008438	1.343966	-1.142566
C	9.652061	0.738550	-0.055692
C	9.652061	-0.738550	0.055692
C	10.053620	-1.580390	-0.987168
C	9.743704	-2.938221	-0.987168
C	-5.440540	8.006763	-0.055692
C	-4.565914	7.881029	-1.142566
C	-3.314836	8.483517	-1.142566
F	9.179032	-5.611521	2.040508
C	8.372294	-5.785984	0.987168
C	7.503928	-6.874880	0.987168
F	7.513356	-7.700213	2.040508
F	4.877610	-7.076630	2.166777
C	4.565914	-7.881029	1.142566
C	3.314836	-8.483517	1.142566
F	2.491592	-8.225675	2.166777
F	-7.513356	-7.700213	2.040508
C	-7.503928	-6.874880	0.987168
C	-8.372294	-5.785984	0.987168
F	-9.179032	-5.611521	2.040508
F	10.110297	-3.677730	-2.040508
C	-9.743704	-2.938221	-0.987168
C	10.053620	-1.580390	-0.987168
F	10.704770	-1.073173	-2.040508
F	10.704770	1.073173	2.040508
C	10.053620	1.580390	0.987168
C	-9.743704	2.938221	0.987168
F	10.110297	3.677730	2.040508
C	8.375761	-4.853283	-0.055692
C	7.533197	-5.119486	-1.142566
C	6.667424	-6.205131	-1.142566
C	6.595383	-7.085807	-0.055692
C	5.440540	-8.006763	0.055692
C	5.032731	-8.845594	-0.987168
C	3.777907	-9.449885	-0.987168
C	2.867819	-9.245721	0.055692
C	-6.595383	-7.085807	-0.055692
C	-6.667424	-6.205131	-1.142566
C	-7.533197	-5.119486	-1.142566
C	-8.375761	-4.853283	-0.055692
C	-9.016651	-3.522462	0.055692
C	-8.699447	-2.697743	1.142566
C	-9.008438	-1.343966	1.142566
C	-9.652061	-0.738550	0.055692
C	-9.652061	0.738550	-0.055692
C	-9.008438	1.343966	-1.142566
C	-8.699447	2.697743	-1.142566
F	-5.813834	-6.330016	-2.166777
F	-7.465009	-4.259508	-2.166777
F	-7.984574	-3.180620	2.166777
F	-8.573872	-0.598738	2.166777
F	-8.573872	0.598738	-2.166777
F	-7.984574	3.180620	-2.166777
F	1.335769	-10.675182	2.040508
C	0.696375	-10.153225	0.987168
C	-0.696375	-10.153225	0.987168
F	-1.335769	-10.675182	2.040508
F	-2.491592	-8.225675	2.166777
C	-3.314836	-8.483517	1.142566
C	-4.565914	-7.881029	1.142566
F	-4.877610	-7.076630	2.166777
C	1.427753	-9.574406	-0.055692
C	0.694296	-9.081638	-1.142566
C	-0.694296	-9.081638	-1.142566
C	-1.427753	-9.574406	-0.055692
C	-2.867819	-9.245721	0.055692
C	-3.777907	-9.449885	-0.987168
C	-5.032731	-8.845594	-0.987168
C	-5.440540	-8.006763	0.055692
F	1.324140	-8.492139	-2.166777
F	-1.324140	-8.492139	-2.166777
F	-3.428302	-10.197575	-2.040508
F	-5.835274	-9.038439	-2.040508
F	5.835274	9.038439	2.040508
F	5.813834	6.330016	2.166777
F	7.465009	4.259508	2.166777
F	3.428302	10.197575	2.040508
F	4.877610	7.076630	-2.166777
F	7.513356	7.700213	-2.040508
F	9.179032	5.611521	-2.040508
F	2.491592	8.225675	-2.166777
C	6.667424	6.205131	1.142566
C	7.533197	5.119486	1.142566
C	3.777907	9.449885	0.987168
C	5.032731	8.845594	0.987168
C	5.440540	8.006763	-0.055692
C	6.595383	7.085807	0.055692
C	7.503928	6.874880	-0.987168
C	8.372294	5.785984	-0.987168

C	9.016651	-3.522462	0.055692	C	-9.016651	3.522462	-0.055692	C	8.375761	4.853283	0.055692
C	-8.375761	4.853283	0.055692	F	7.465009	-4.259508	-2.166777	C	2.867819	9.245721	-0.055692
C	-8.372294	5.785984	-0.987168	F	5.813834	-6.330016	-2.166777	C	3.314836	8.483517	-1.142566
C	-7.503928	6.874880	-0.987168	F	5.835274	-9.038439	-2.040508	C	4.565914	7.881029	-1.142566
C	-6.595383	7.085807	0.055692	F	3.428302	-10.197575	-2.040508				

PF[16]CPP

C	-10.463787	0.694381	1.133352	C	10.923432	3.771167	0.997239	F	-5.849699	-10.630857	2.048553
C	-10.962613	1.427184	0.049046	C	10.390333	5.057418	0.997118	F	-3.381500	-11.653922	2.048741
C	-11.535376	0.696184	-0.996965	C	9.581315	5.513328	-0.048906	F	-2.554477	-9.634569	-2.158557
C	-11.535381	-0.696168	-0.996979	C	9.400934	4.645255	-1.133078	F	-5.005249	-8.619030	-2.158877
C	-10.962619	-1.427192	0.049019	C	9.932640	3.362268	-1.132942	F	-1.326349	-9.879244	2.159110
C	-10.463795	-0.694412	1.133342	C	8.760220	6.741902	0.048497	F	1.326364	-9.879237	2.159096
C	-10.674136	-2.876448	-0.048647	C	8.648144	7.663622	-0.997645	F	1.335893	-12.060759	-2.048563
C	-10.923432	-3.771167	0.997239	C	7.663625	8.648176	-0.997643	F	-1.335915	-12.060744	-2.048557
C	-10.390333	-5.057418	0.997118	C	6.741906	8.760273	0.048500	F	3.381517	-11.653971	2.048694
C	-9.581315	-5.513328	-0.048906	C	6.907410	7.889470	1.132859	F	5.849712	-10.630895	2.048519
C	-9.400934	-4.645255	-1.133078	C	7.889423	6.907430	1.132858	F	5.005227	-8.618946	-2.158845
C	-9.932640	-3.362268	-1.132942	C	5.513339	9.581379	-0.048903	F	2.554450	-9.634465	-2.158527
C	-8.760220	-6.741902	0.048497	C	5.057409	10.390361	0.997140	F	6.047367	-7.923137	2.158802
C	-7.889423	-6.907430	1.132858	C	3.771151	10.923442	0.997266	F	7.923157	-6.047371	2.158809
C	-6.907410	-7.889470	1.132859	C	2.876446	10.674159	-0.048632	F	9.471914	-7.582680	-2.049231
C	-6.741906	-8.760273	0.048500	C	3.362281	9.932699	-1.132943	F	7.582669	-9.471901	-2.049235
C	-7.663625	-8.648176	-0.997643	C	4.645275	9.401013	-1.133087	F	10.630829	-5.849689	2.048599
C	-8.648144	-7.663622	-0.997645	C	1.427188	10.962637	0.049016	F	8.619025	-5.005260	-2.158846
C	-5.513339	-9.581379	-0.048903	C	0.696181	11.535391	-0.996995	F	9.634558	-2.554485	-2.158528
C	-5.057409	-10.390361	0.997140	C	-0.696170	11.535394	-0.997001	F	11.653889	-3.381491	2.048785
C	-3.771151	-10.923442	0.997266	C	-1.427187	10.962640	0.049006	F	12.060736	-1.335923	-2.048520
C	-2.876446	-10.674159	-0.048632	C	-0.694401	10.463824	1.133329	F	12.060754	1.335882	-2.048542
C	-3.362281	-9.932699	-1.132943	C	0.694393	10.463818	1.133331	F	9.879199	1.326383	2.159100
C	-4.645275	-9.401013	-1.133087	C	-2.876444	10.674153	-0.048650	F	9.879201	-1.326330	2.159129
C	-1.427188	-10.962637	0.049016	C	-3.771159	10.923458	0.997238	F	11.653931	3.381528	2.048706
C	-0.694393	-10.463818	1.133331	C	-5.057408	10.390356	0.997127	F	10.630861	5.849724	2.048512
C	0.694401	-10.463824	1.133329	C	-5.513320	9.581327	-0.048888	F	8.618944	5.005225	-2.158864
C	1.427187	-10.962640	0.049006	C	-4.645252	9.400941	-1.133064	F	9.634457	2.554446	-2.158528
C	0.696170	-11.535394	-0.997001	C	-3.362267	9.932650	-1.132938	F	9.471899	7.582670	-2.049263
C	-0.696181	-11.535391	-0.996995	C	9.581359	-5.513340	-0.048863	F	7.582683	9.471922	-2.049269
C	2.876444	-10.674153	-0.048650	C	-7.663616	8.648149	-0.997614	F	6.047368	7.923190	2.158778
C	3.771159	-10.923458	0.997238	C	-6.741892	8.760230	0.048524	F	7.923130	6.047394	2.158781
C	5.057408	-10.390356	0.997127	C	-6.907411	7.889431	1.132885	F	5.849699	10.630857	2.048553
C	5.513320	-9.581327	-0.048888	C	-7.889447	6.907415	1.132891	F	3.381500	11.653922	2.048741
C	4.645252	-9.400941	-1.133064	C	-8.760257	6.741908	0.048537	F	2.554477	9.634569	-2.158557
C	3.362267	-9.932650	-1.132938	C	-8.648167	7.663627	-0.997606	F	5.005249	8.619030	-2.158877
C	6.741892	-8.760230	0.048524	C	-9.581359	5.513340	-0.048863	F	1.335915	12.060744	-2.048557
C	6.907411	-7.889431	1.132885	C	-10.390334	5.057403	0.997182	F	-1.335893	12.060759	-2.048563
C	7.889447	-6.907415	1.132891	C	-10.923413	3.771145	0.997307	F	-1.326364	9.879237	2.159096
C	8.760257	-6.741908	0.048537	C	-9.932684	3.362285	-1.132909	F	1.326349	9.879244	2.159110
C	8.648167	-7.663627	-0.997606	C	-9.400999	4.645280	-1.133051	F	-3.381517	11.653971	2.048694
C	7.663616	-8.648149	-0.997614	C	-10.674136	2.876442	-0.048596	F	-5.849712	10.630895	2.048519
C	10.390334	-5.057403	0.997182	F	-9.879201	1.326330	2.159129	F	-5.005227	8.618946	-2.158845
C	9.400999	-4.645280	-1.133051	F	-12.060736	1.335923	-2.048520	F	-2.554450	9.634465	-2.158527
C	9.932684	-3.362285	-1.132909	F	-12.060754	-1.335882	-2.048542	F	-7.582669	9.471901	-2.049235
C	10.674136	-2.876442	-0.048596	F	-9.879199	-1.326383	2.159100	F	-6.047367	7.923137	2.158802
C	10.923413	-3.771145	0.997307	F	-11.653931	-3.381528	2.048706	F	-7.923157	6.047371	2.158809
C	10.962613	-1.427184	0.049046	F	-10.630861	-5.849724	2.048512	F	-9.471914	7.582680	-2.049231
C	11.535376	-0.696184	-0.996965	F	-8.618944	-5.005225	-2.158864	F	-10.630829	5.849689	2.048599
C	11.535381	0.696168	-0.996979	F	-9.634457	-2.554446	-2.158528	F	-11.653889	3.381491	2.048785
C	10.962619	1.427192	0.049019	F	-7.923130	-6.047394	2.158781	F	-9.634558	2.554485	-2.158528
C	10.463795	0.694412	1.133342	F	-6.047368	-7.923190	2.158778	F	-8.619025	5.005260	-2.158846
C	10.463787	-0.694381	1.133352	F	-7.582683	-9.471922	-2.049269				
C	10.674136	2.876448	-0.048647	F	-9.471899	-7.582670	-2.049263				

Perfluorobiphenyl

C	-1.468854	1.063379	0.544907	C	1.468853	1.063379	-0.544907	F	3.528233	2.104716	-1.075261
C	-2.860345	1.075862	0.545190	C	0.739875	0.000000	0.000000	F	0.827233	2.100682	-1.095999
C	-2.860344	-1.075862	-0.545190	C	1.468853	-1.063379	0.544906	F	-0.827232	2.100681	1.095999

C	-1.468853	-1.063379	-0.544906	C	2.860344	-1.075863	0.545190	F	-3.528233	2.104716	1.075261
F	-0.827232	-2.100681	-1.095998	C	3.557547	0.000000	0.000001	F	-4.891049	0.000000	-0.000001
F	-3.528232	-2.104717	-1.075260	F	3.528232	-2.104717	1.075260	F	4.891049	0.000000	0.000001
C	-3.557547	0.000000	-0.000001	F	0.827231	-2.100681	1.095998				
C	2.860345	1.075862	-0.545191	C	-0.739875	0.000000	0.000000				

Perfluoro-*p*-terphenyl

C	-0.695164	0.924628	-0.753495	C	-5.025386	-1.168763	-0.298328	C	5.025390	1.168734	0.298436
C	0.695155	0.924633	-0.753493	C	-5.722447	0.000000	0.000000	C	5.722452	0.000000	0.000000
C	0.695155	-0.924633	0.753492	F	-5.693366	-2.287904	-0.592660	C	5.025390	-1.168734	-0.298436
C	-0.695163	-0.924629	0.753496	F	-2.992619	-2.288832	-0.613250	C	3.633934	-1.157015	-0.300020
F	-1.335218	-1.820755	1.515571	C	-1.425825	0.000000	0.000000	F	2.992634	-2.288768	-0.613512
F	1.335200	-1.820741	1.515599	F	-5.693366	2.287904	0.592660	F	5.693369	-2.287845	-0.592886
C	1.425821	0.000000	0.000000	F	-2.992619	2.288832	0.613250	F	7.055793	0.000000	0.000001
C	-5.025386	1.168763	0.298328	F	-1.335218	1.820755	-1.515571	F	5.693369	2.287845	0.592886
C	-3.633930	1.157041	0.299918	F	1.335199	1.820741	-1.515600	F	2.992633	2.288768	0.613511
C	-2.905274	0.000000	0.000000	C	2.905272	0.000000	0.000000	F	-7.055789	0.000000	0.000000
C	-3.633930	-1.157041	-0.299917	C	3.633934	1.157015	0.300020				

8. NMR spectra and HRMS of PFCPPs

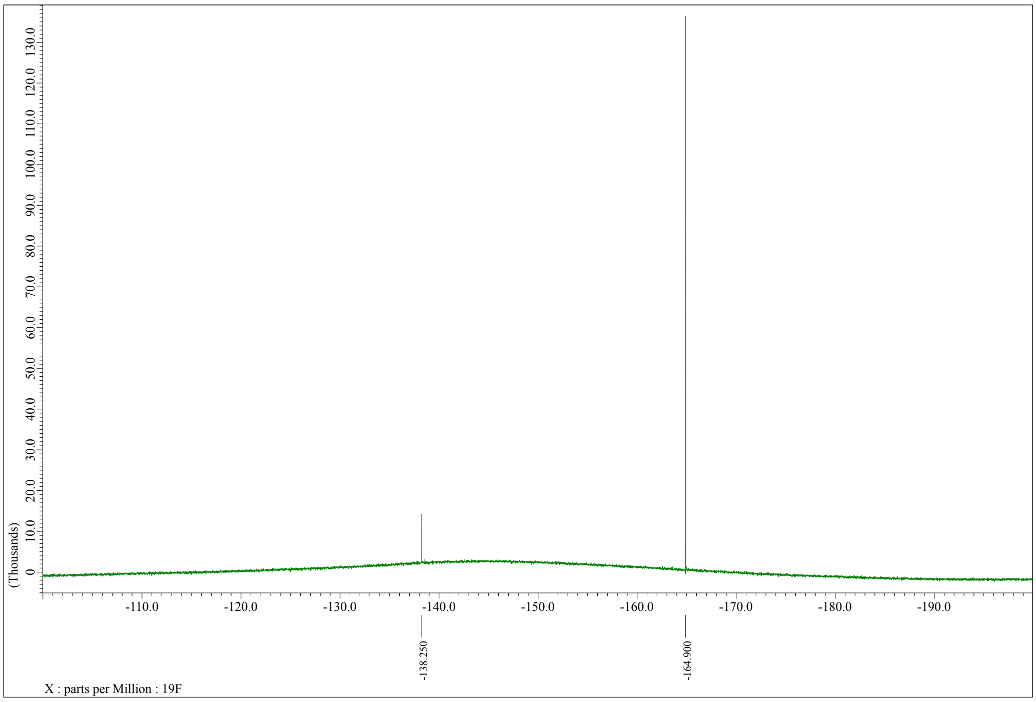

Supplementary Fig. 16. ^{19}F NMR spectrum of **PF[10]CPP** (565 MHz, $CDCl_3$). Reference: C_6F_6 (–164.9 ppm)

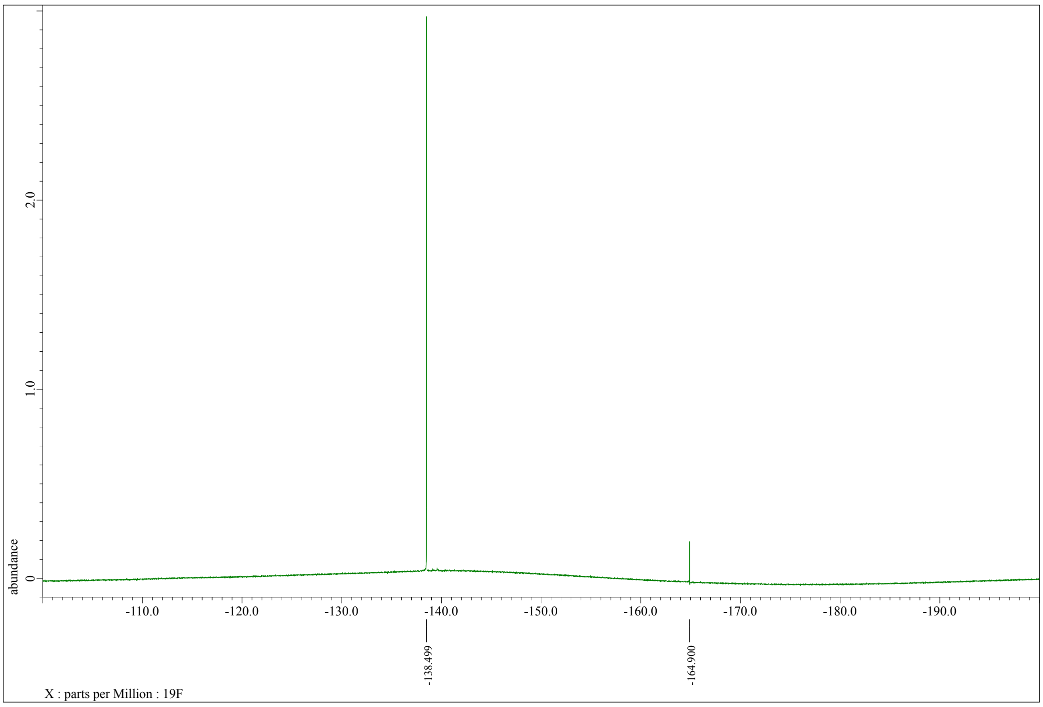

Supplementary Fig. 17. ^{19}F NMR spectrum of **PF[12]CPP** (565 MHz, $CDCl_3$). Reference: C_6F_6 (–164.9 ppm)

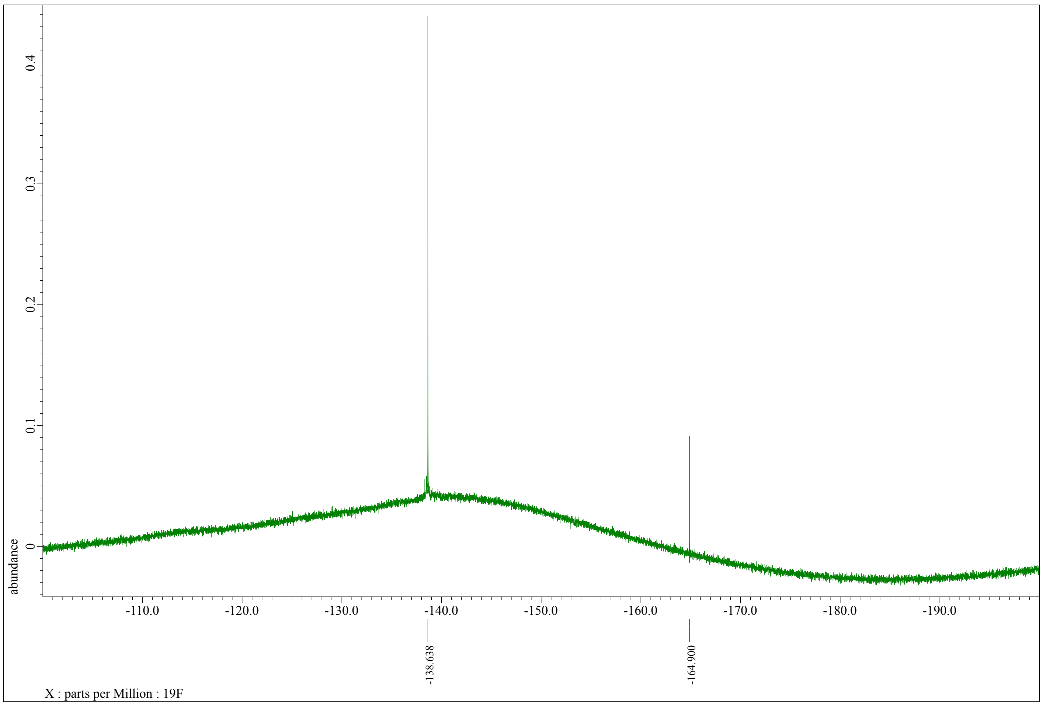

Supplementary Fig. 18. ^{19}F NMR spectrum of **PF[14]CPP** (565 MHz, $CDCl_3$). Reference: C_6F_6 (–164.9 ppm)

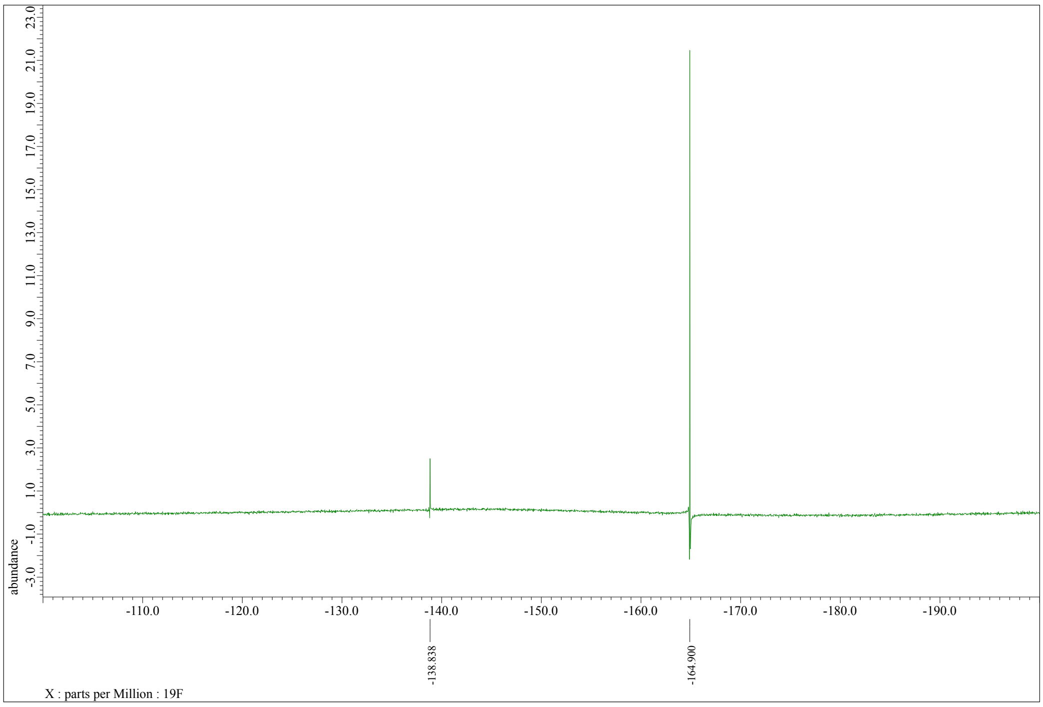

Supplementary Fig. 19. ^{19}F NMR spectrum of **PF[16]CPP** (565 MHz, $CDCl_3$). Reference: C_6F_6 (–164.9 ppm)

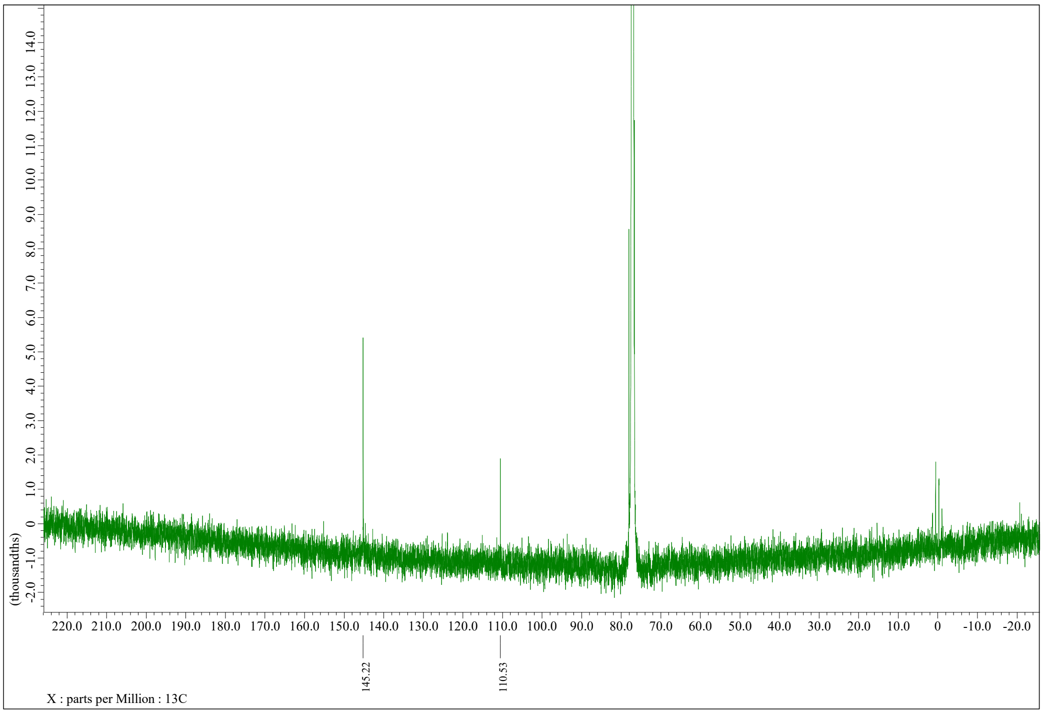

Supplementary Fig. 20. $^{13}C\{^{19}F\}$ NMR spectrum of **PF[10]CPP** (150 MHz, $CDCl_3$).

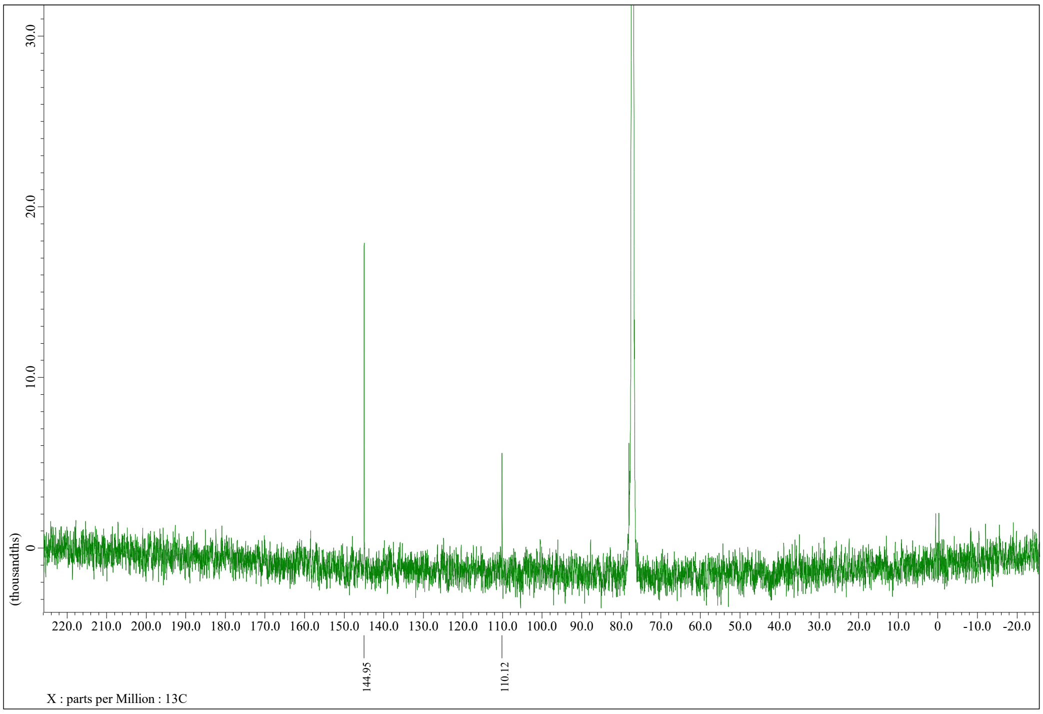

Supplementary Fig. 21. $^{13}C\{^{19}F\}$ NMR spectrum of **PF[12]CPP** (150 MHz, $CDCl_3$).

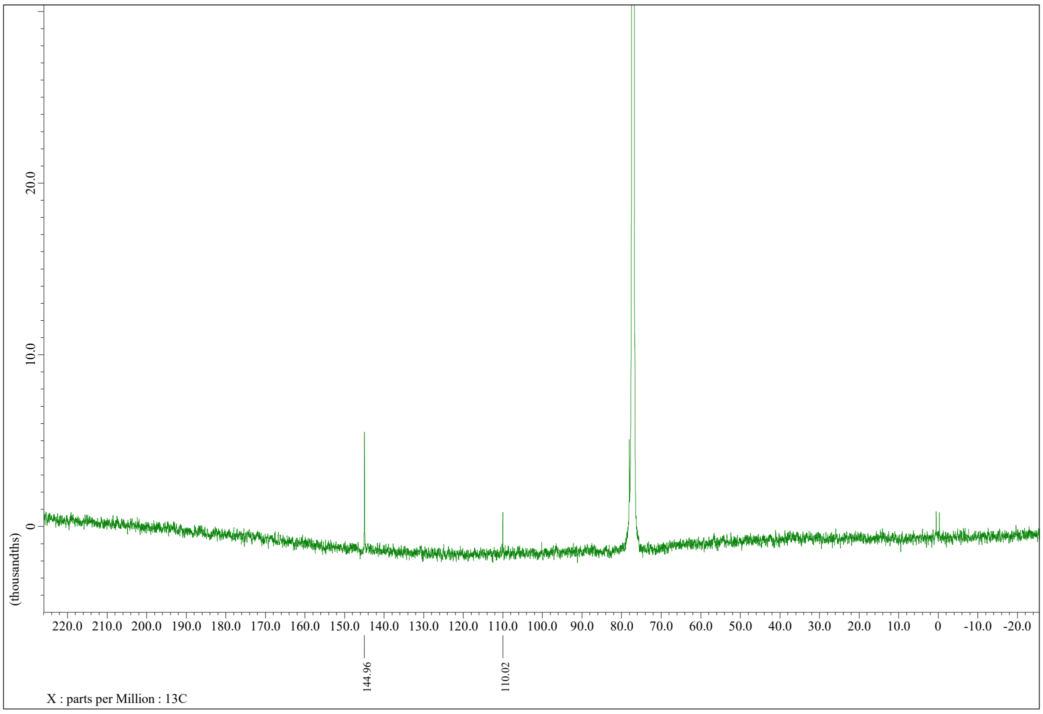

Supplementary Fig. 22. $^{13}C\{^{19}F\}$ NMR spectrum of **PF[14]CPP** (150 MHz, $CDCl_3$, 50 °C).

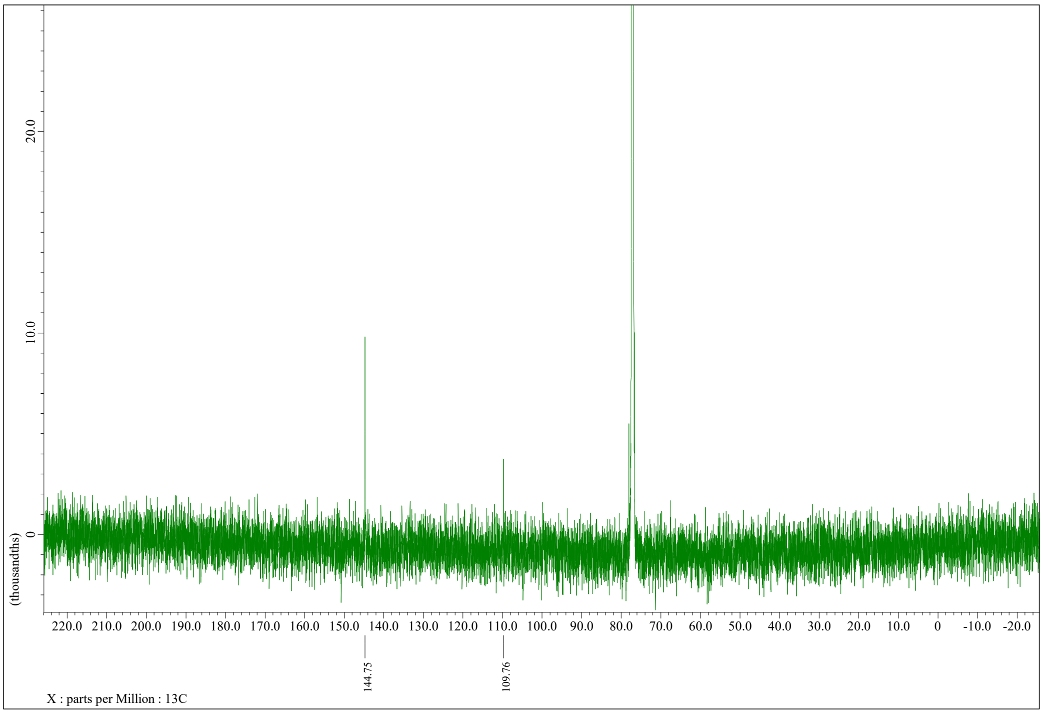

Supplementary Fig. 23. $^{13}C\{^{19}F\}$ NMR spectrum of **PF[16]CPP** (150 MHz, $CDCl_3$).

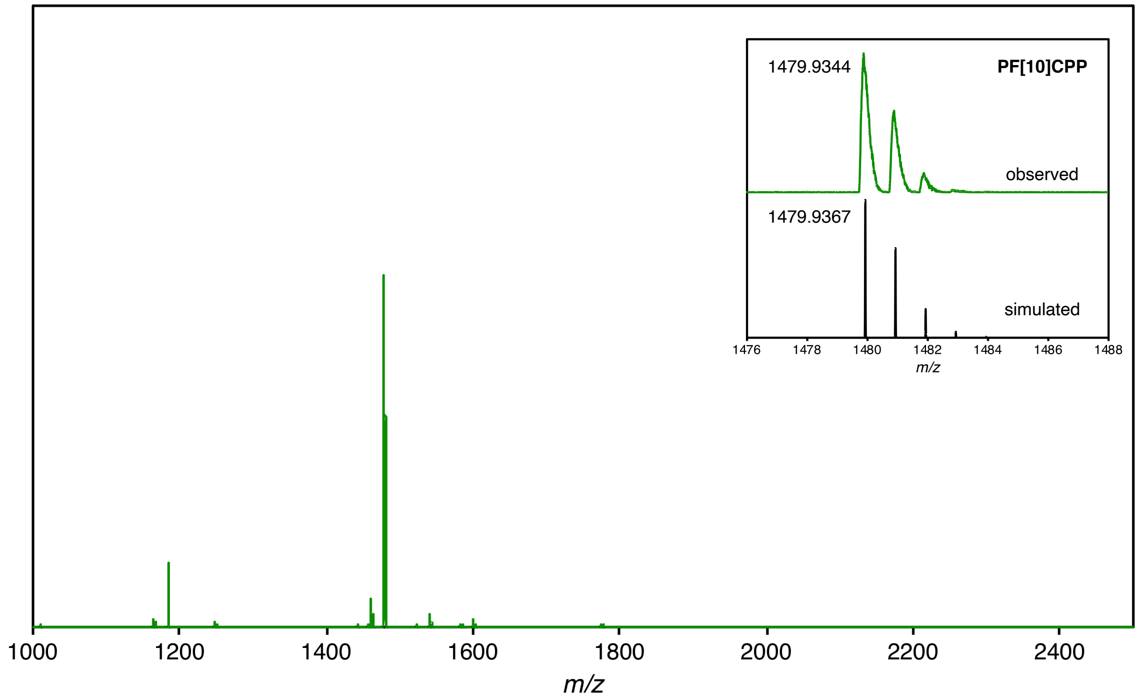

Supplementary Fig. 24. LDI-TOF mass spectrum of **PF[10]CPP**.

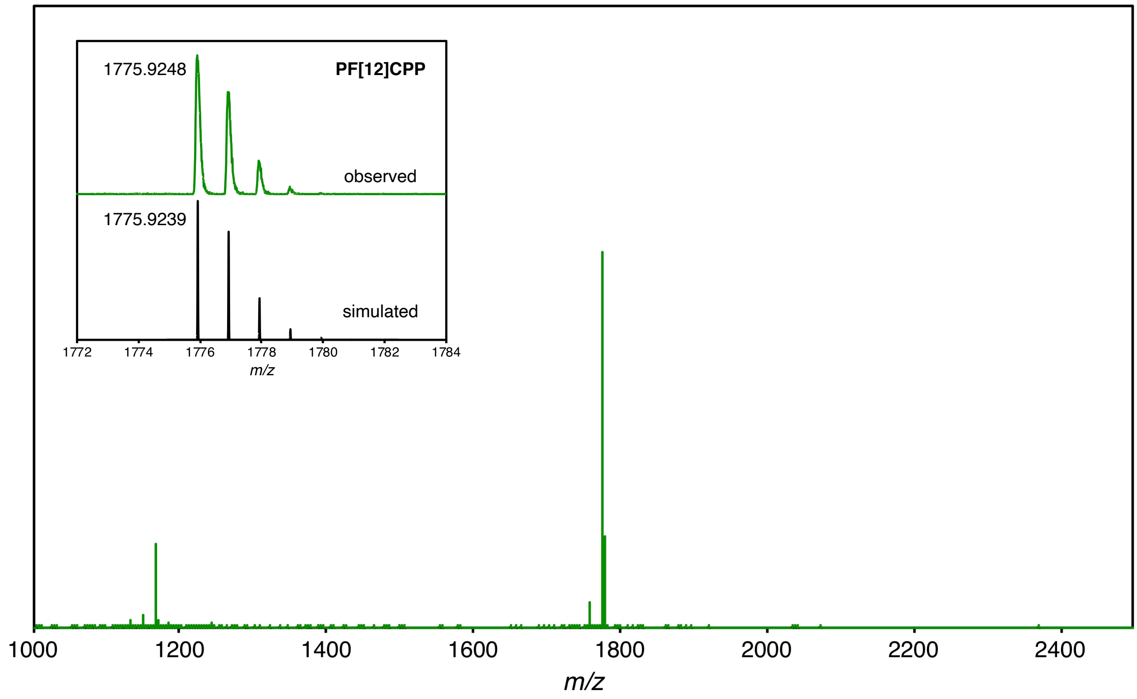

Supplementary Fig. 25. LDI-TOF mass spectrum of **PF[12]CPP**.

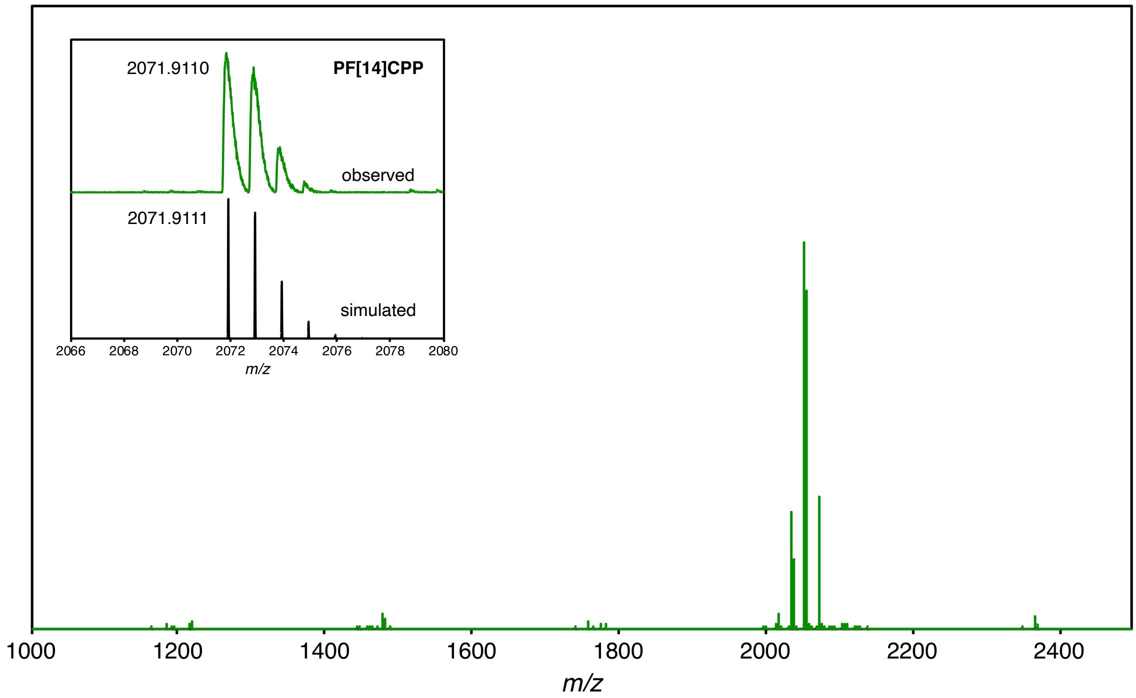

Supplementary Fig. 26. LDI-TOF mass spectrum of **PF[14]CPP**.

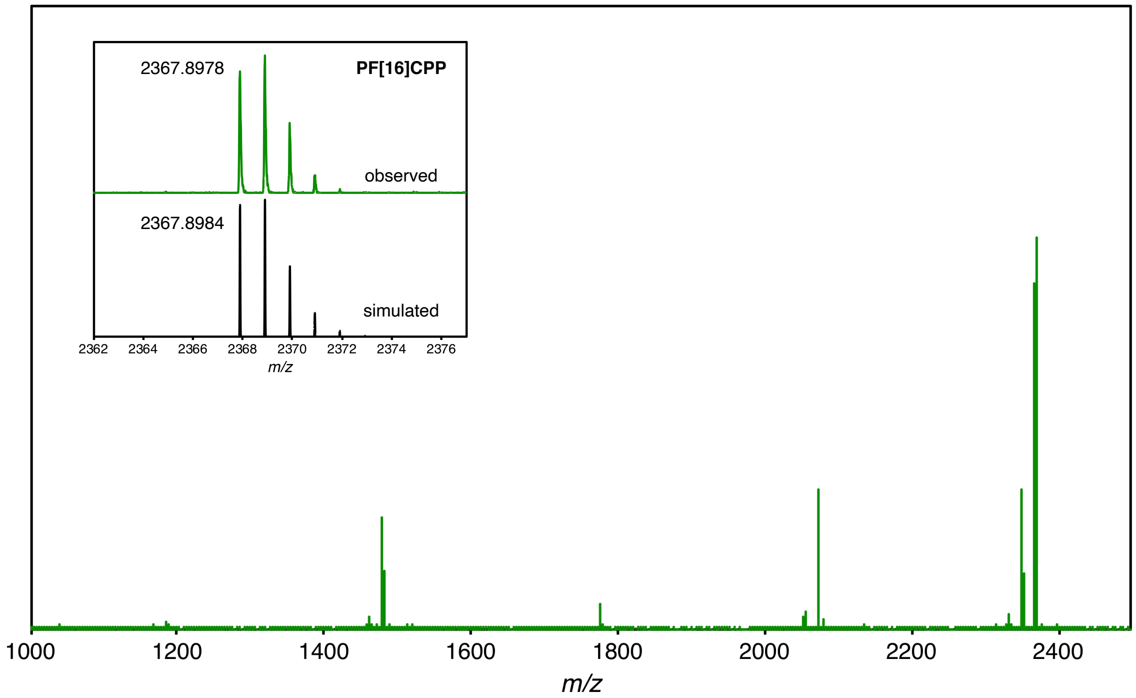

Supplementary Fig. 27. LDI-TOF mass spectrum of **PF[16]CPP**.

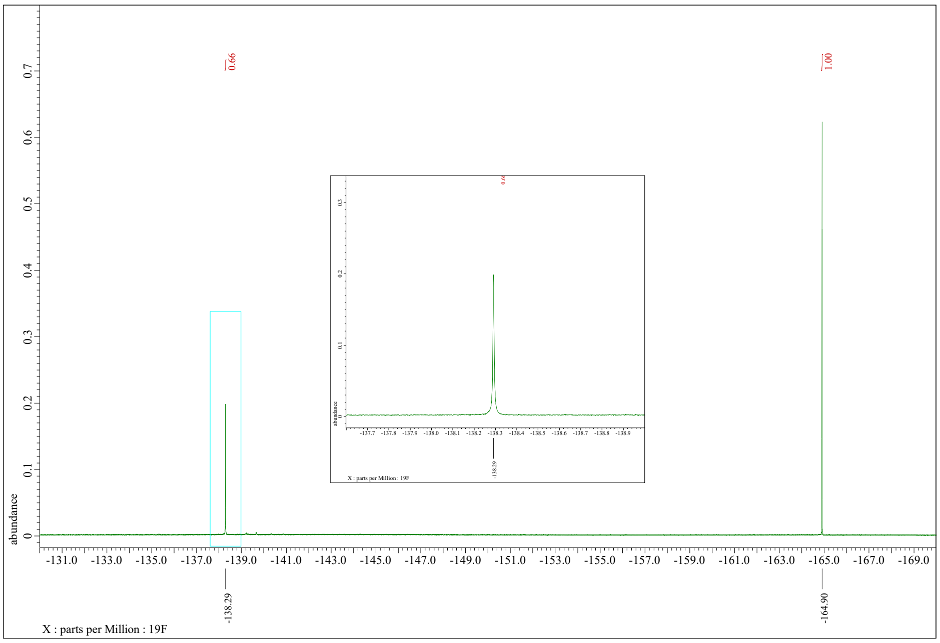

Supplementary Fig. 28. ^{19}F NMR spectrum of **PF[10]CPP** (565 MHz, $CDCl_3$) for C_{60} encapsulation experiment. Reference: C_6F_6 (–164.9 ppm).

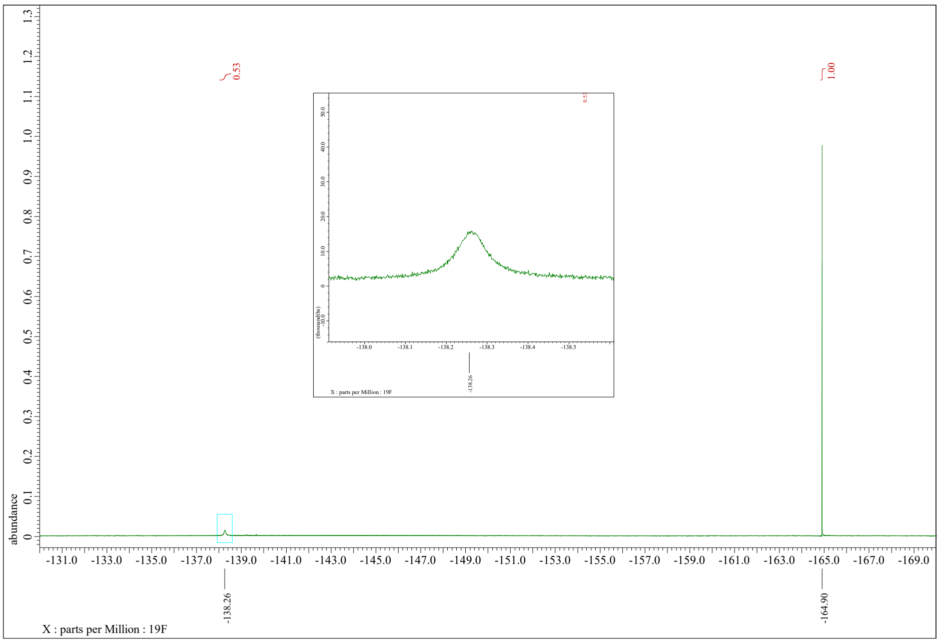

Supplementary Fig. 29. ^{19}F NMR spectrum of **PF[10]CPP** (565 MHz, $CDCl_3$) with 9 equiv. of C_{60}. Reference: C_6F_6 (–164.9 ppm).

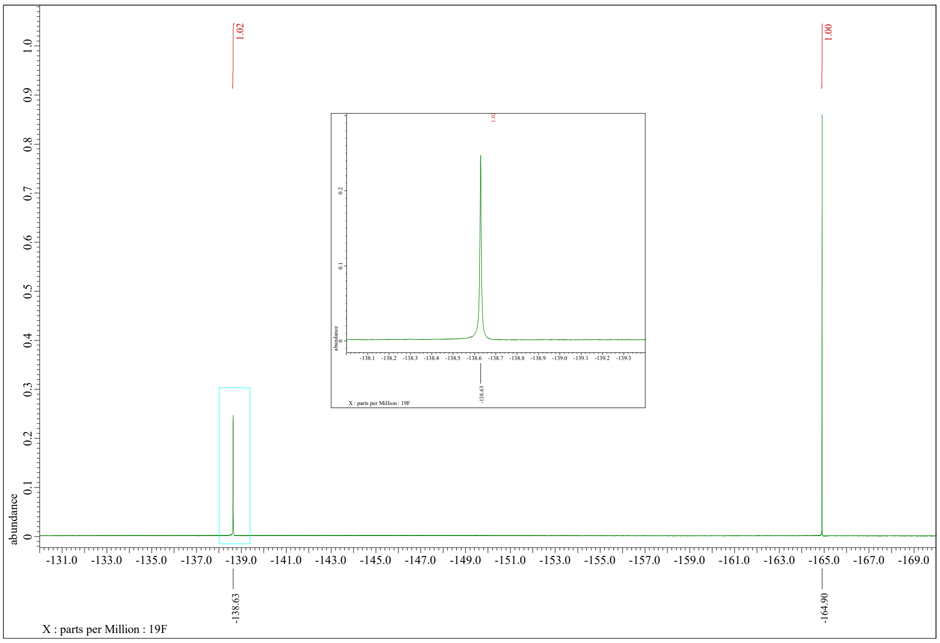

Supplementary Fig. 30. ^{19}F NMR spectrum of **PF[12]CPP** (565 MHz, $CDCl_3$) for C_{60} encapsulation experiment. Reference: C_6F_6 (–164.9 ppm).

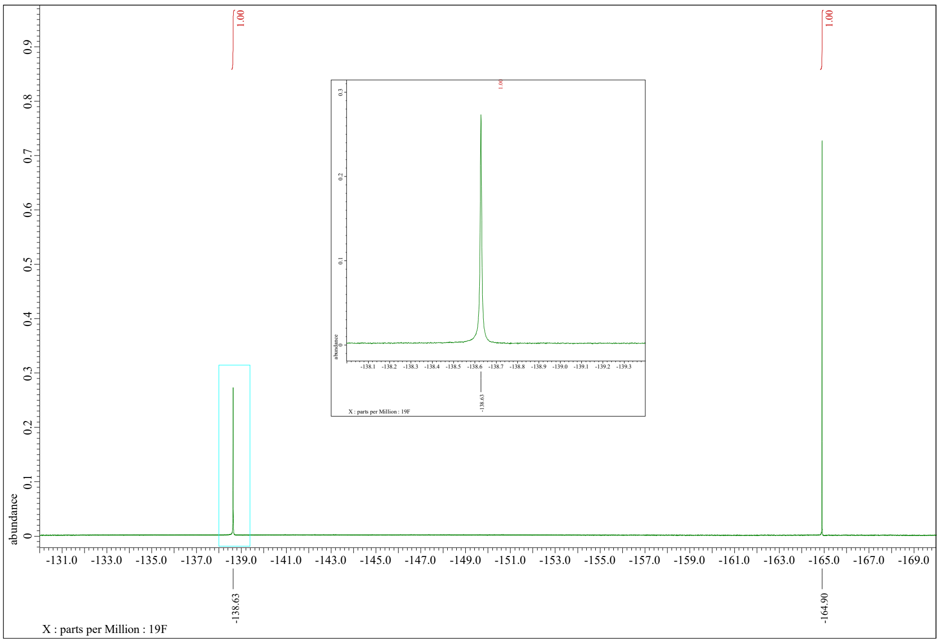

Supplementary Fig. 31. ^{19}F NMR spectrum of **PF[12]CPP** (565 MHz, $CDCl_3$) with 13 equiv. of C_{60}. Reference: C_6F_6 (–164.9 ppm).

Supplementary References

1 Sheldrick, G. M. SHELXT - integrated space-group and crystal-structure determination. *Acta Crystallogr.* **A71**, 3–8 (2015).

2 Sheldrick, G. M. Crystal structure refinement with SHELXL. *Acta Crystallogr.* **C71**, 3–8 (2015).

3 Dolomanov, O. V., Bourhis, L. J., Gildea, R. J., Howard, J. A. K. & Puschmann, H. Olex2: A complete structure solution, refinement and analysis program. *J. Appl. Crystallogr.* **42**, 339–341 (2009).

4 Segawa, Y. *et al.* Combined experimental and theoretical studies on the photophysical properties of cycloparaphenylenes. *Org. Biomol. Chem.* **10**, 5979–5984 (2012).

5 Ishii, Y., Matsuura, S., Segawa, Y. & Itami, K. Synthesis and Dimerization of Chloro[10]Cycloparaphenylene: A Directly Connected Cycloparaphenylene Dimer. *Org. Lett.* **16**, 2174–2176 (2014).

6 Frisch, M. J. *et al.* Gaussian 16, Revision B.01, Gaussian, Inc., Wallingford CT, 2016.

7 Becke, A. D. Density functional thermochemistry. III. The role of exact exchange. *J. Chem. Phys.* **98**, 5648–5652 (1993).

8 Lee, C., Yang, W. & Parr, R. G. Development of the Colle-Salvetti correlation-energy formula into a functional of the electron density. *Phys. Rev. B* **37**, 785–789 (1988).

9 Segawa, Y., Omachi, H. & Itami, K. Theoretical studies on the structures and strain energies of cycloparaphenylenes. *Org. Lett.* **12**, 2262–2265 (2010).
