# Supplementary material for: Perfluorocycloparaphenylenes
Source: Nat Commun. 2022 Jun 28;13:3713. doi: 10.1038/s41467-022-31530-x (PMC9240036; doi:10.1038/s41467-022-31530-x)
Supplement: Supplementary file 2 — Description of Additional Supplementary Files [file 41467_2022_31530_MOESM2_ESM.pdf]

Supplementary Movie 1: 3-dimensional rendering of the villus tip with a VTT highlighted (green).

Supplementary Data 1: UMAP cluster-specific genes from intestinal fibroblast single cell RNA sequencing (Fig. 4b).

Supplementary Data 2: UMAP cluster-specific genes from re-clustering of subepithelial B cluster (Extended Data Fig. 6b).

Supplementary Data 3: Portal and tail vein blood plasma metabolomics data from [U-^{13}C]-glucose gavaged control and *Adamts18*$^{-/-}$ mice.

Supplementary Data 4: Analysis for fractions of [U-^{13}C]-labeled metabolites from [U-^{13}C]-glucose gavaged control and *Adamts18*$^{-/-}$ mice.

Supplementary Data 5: Portal vein blood plasma metabolomics data from starved control and *Adamts18*$^{-/-}$ mice. Significance was calculated using Student's *t* test and corrected for multiple hypothesis testing with the BH method.

Supplementary Data 6: Tail vein blood plasma metabolomics data from protein-fed control and *Adamts18*$^{-/-}$ mice.

Supplementary Data 7: Metabolic pathway analysis of portal vein blood plasma metabolites from starved control and *Adamts18*$^{-/-}$ mice. Significance was calculated using Student's *t* test and corrected for multiple hypothesis testing with the BH method.
